# Conformational Dynamics and Catalytic Backups in a Hyper-Thermostable Engineered Archaeal Protein Tyrosine Phosphatase

**DOI:** 10.1101/2025.03.26.645524

**Published:** 2025-03-26

**Authors:** Dariia Yehorova, Nikolas Alansson, Ruidan Shen, Joshua M. Denson, Michael Robinson, Valeria A. Risso, Nuria Ramirez Molina, J. Patrick Loria, Eric A. Gaucher, Jose M. Sanchez-Ruiz, Alvan C. Hengge, Sean J. Johnson, Shina C. L. Kamerlin

**Affiliations:** 1. School of Chemistry and Biochemistry, Georgia Institute of Technology, 901 Atlantic Drive NW, Atlanta, GA 30332, USA; 2. Department of Chemistry & Biochemistry, Utah State University, 0300 Old Main Hill, Logan, UT 84322-0300, USA; 3. Department of Chemistry – BMC, Uppsala University, BMC Box 576, S-751 23 Uppsala, Sweden; 4. Departamento de Química Física, Facultad de Ciencias, Unidad de Excelencia de Química Aplicada a Biomedicina y Medioambiente (UEQ), Universidad de Granada, Granada, 18071, Spain; 5. Department of Biology, Georgia State University, 100 Piedmont Ave SE, Atlanta, GA 30303, USA; 6. Department of Chemistry, Yale University, P. O. Box 208107, New Haven, CT, 06520-8107; 7. Department of Molecular Biophysics and Biochemistry, Yale University, New Haven, CT, 06520, USA; 8. Department of Chemistry, Lund University, Box 124, 22100 Lund, Sweden

**Keywords:** archaea, protein tyrosine phosphatase, sequence shuffling, catalytic backups, protein conformational dynamics

## Abstract

Protein tyrosine phosphatases (PTPs) are a family of enzymes that play important roles in regulating cellular signaling pathways. The activity of these enzymes is regulated by the motion of a catalytic loop that places a critical conserved aspartic acid side chain into the active site for acid-base catalysis upon loop closure. These enzymes also have a conserved phosphate binding loop that is typically highly rigid and forms a well-defined anion binding nest. The intimate links between loop dynamics and chemistry in these enzymes make PTPs an excellent model system for understanding the role of loop dynamics in protein function and evolution. In this context, archaeal PTPs, which have evolved in extremophilic organisms, are highly understudied, despite their unusual biophysical properties. We present here an engineered chimeric PTP (ShufPTP) generated by shuffling the amino acid sequence of five extant hyperthermophilic archaeal PTPs. Despite ShufPTP’s high sequence similarity to its natural counterparts, ShufPTP presents a suite of unique properties, including high flexibility of the phosphate binding P-loop, facile oxidation of the active site cysteine, mechanistic promiscuity, and most notably, hyperthermostability, with a denaturation temperature likely >130 °C (>8°C higher than the highest recorded growth temperature of any archaeal strain). Our combined structural, biochemical, biophysical and computational analysis provides insight both into how small steps in evolutionary space can radically modulate the biophysical properties of an enzyme, and showcase the tremendous potential of archaeal enzymes for biotechnology, to generate novel enzymes capable of operating under extreme conditions.

## Graphical Abstract

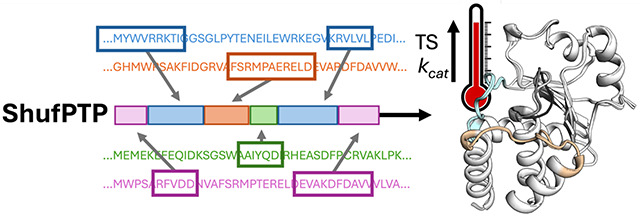

## Introduction

Enzymes are exquisite catalysts,^1^ and there remains tremendous interest in exploiting tailored enzymes for use in biotechnology and beyond.^2–5^ While there has been significant progress in enzyme engineering, major leaps forward will require innovation. In this context, there is increasing evidence that conformational dynamics plays an important role in the emergence of new enzyme activities, and in the evolutionary optimization of enzyme selectivity and activity.^6–10^ In particular, enzyme active site loops are highly flexible, and evidence shows that evolutionary conformational modulation of their dynamical behavior can translate into control of enzyme specificity, activity, and even the pH dependency of catalysis.^11^ There exist numerous illustrations of such evolutionarily modulated loop dynamics being functionally important, spanning a wide range of enzymes catalyzing chemically distinct reactions,^36^ suggesting that this is a generalizable principle. Understanding how active site loop dynamics is evolutionarily and allosterically regulated, and how it is linked to the chemical step of catalysis, is thus important for our understanding of the factors shaping new enzyme functions and activities more broadly. Further, there is increasing evidence that such loop dynamics can also be artificially exploited in protein engineering to control protein activity, selectivity, and biophysical properties.^11, 12^

**Figure 1.**
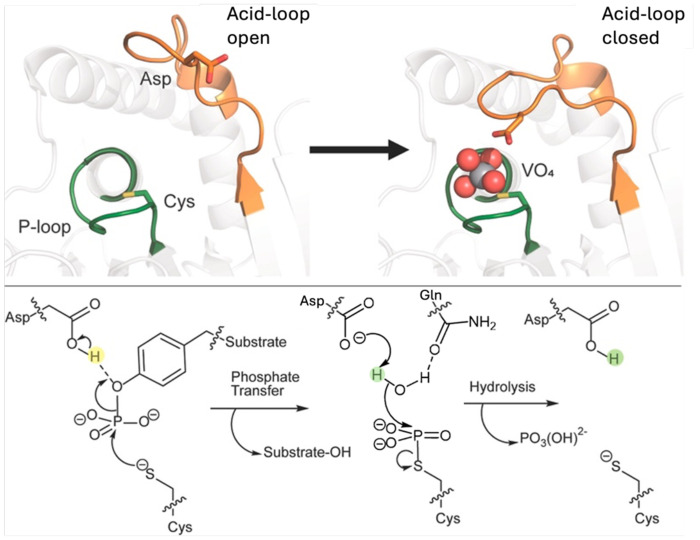
Overview of the PTP catalytic mechanism. Two-step mechanism catalyzed by PTPs, illustrating acidloop conformational changes, as well as the position of the catalytic aspartic acid. From Ref. ^19^, published by the American Chemical Society, under a CC-BY license. Copyright © 2024 The Authors.

Protein tyrosine phosphatases are an excellent model system for probing the links between loop dynamics and the evolution of enzyme activity.^1–33, 37, 38^ These genetically diverse enzymes^13^ share a common core structure, chemical mechanism, and enzymatic transition states.^14^ Yet, their catalytic rates vary by orders of magnitude,^15^ reflecting the variety of regulatory roles they play *in vivo*. Structurally, PTPs share a number of catalytic loops that decorate their active sites (Figure 1): the highly conserved phosphate-binding P-loop; a highly mobile acid-loop (the WPD/IPD loop), which carries a critical aspartic acid, and undergoes a substantial conformational change (~10Å) between catalytically inactive “open” and active “closed” conformations; as well as additional Q- and E-loops, which carry catalytically and structurally important residues.^16^ From a dynamical perspective, is noteworthy that, unlike many enzymes that are regulated by catalytic loop motion, the acid-loop of PTPs does not form a lid over the active site. Rather, it plays a key chemical role, positioning the conserved Asp side chain on the loop in an optimal position for catalysis (Figure 1). Thus, unlike say TIM-barrel proteins (which are frequently decorated by mobile catalytic loops^17^), the PTP acid-loop does not form an active site cage, but rather the role of loop motion is primarily chemical, and substrate/product can diffuse in and out of the active site from an acid-loop closed position.^18^

In the best characterized PTPs, PTP1B^20^ and YopH,^21^ burst kinetics indicate that hydrolysis is rate-limiting. However, NMR studies of these PTPs have shown correlation between the rates of loop motion and phosphotyrosine cleavage kinetics.^22^ This has been supported by computational, biochemical and structural characterization, suggesting a direct link between loop dynamics, turnover rates, and pH-dependency, in wild-type PTP1B and YopH and variants.^15, 16, 22–26^ Furthermore, computational studies indicate that the acid-loop of PTPs is conformationally plastic, sampling a diversity of open conformations.^16, 26^ This is important in the context of evidence that suggests that increased activity among the PTP superfamily is directly linked to destabilization of the acid-loop open conformation, while stabilizing the catalytically active loop-closed conformation.^27^

**Figure 2.**
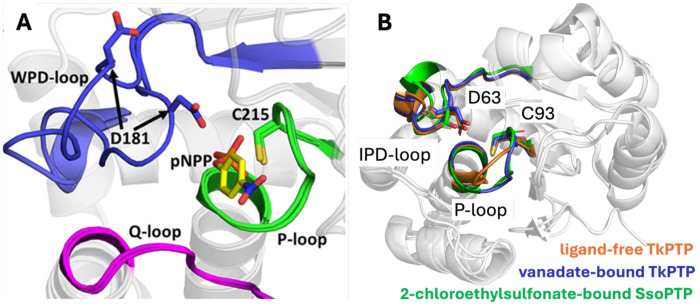
Key loops decorating PTP active sites. (**A**) A comparison of the open and closed conformations of the acid-loop of the archetypal member of the protein tyrosine phosphatase superfamily, PTP1B, highlighting also the Q- and P-loops. (**B**) An overlay of the IPD- and P-loops of the archaeal PTPs, SsoPTP (PDB ID: 7MPD^32^) and TkPTP (PDB IDs: 5Z5A and 5Z59^33^), in both their unliganded and liganded forms. In both archaeal PTPs, the acid-loop is closed and in the same position in all structures, but the phosphate binding P-loop is flexible and takes on multiple conformations (note that only one conformation of SsoPTP’s P-loop could be captured structurally, the existence of the other was determined through NMR spectroscopy^32^). Panel A was originally published in ref. ^16^ under a CC-BY license. Published by the American Chemical Society. Copyright © 2021 The Authors.

Curiously, while the phosphate-binding P-loop is relatively rigid and structurally conserved in many enzymes,^16, 28–32^ archaeal PTPs are a notable exception, with evidence for temperature-dependent transitions between active and inactive conformations (Figure 2).^32, 33^ Additionally, while archaeal PTPs are far less characterized than their eukaryotic and prokaryotic counterparts, evolutionary studies suggest that the phosphorylation/dephosphorylation machinery of eukaryotes and prokaryotes evolved from archaea.^34, 35^ Therefore, better understanding of the unusual dynamical properties of catalytic loops in archaeal PTPs will aid in understanding the links between loop dynamics and catalysis in the PTP superfamily more broadly, and in loopy enzymes more generally.^12^

DNA shuffling is a well-established evolutionary protein engineering technique that can be used to expand protein functional diversity, and/or manipulate protein biophysical properties, and has a well-established and distinguished history as a tool for protein engineering.^33, 36–38^ Here, we have applied amino acid shuffling to five thermophilic archaeal PTP sequences, and in doing so have generated a synthetic chimeric archaeal PTP, ShufPTP, with highly unusual biochemical and biophysical properties. Specifically, ShufPTP shares 90% sequence similarity with its closest extant counterpart, a PTP from the thermophilic anaerobic archaeon, *Thermococcus gorgonarius* (Genbank ID: WP_088884773.1, TgPTP), from a family of archaea typically found in hydrothermal vents.^39^ Computational, structural, biochemical and biophysical characterization shows that, curiously, ShufPTP is (1) hyperthermostable (*T*_M_ > 130 °C), (2) has a mobile phosphate-binding P-loop that takes on multiple configurations, (3) is mechanistically promiscuous, exploiting a backup catalytic mechanism, and (4) has a nucleophilic cysteine that is readily amenable to oxidation, a property that has been suggested to be important for the regulation of PTP activity.^40–46^ Glimmers of each of these properties have been seen in extant archaeal PTPs such as TkPTP^33^ and SsoPTP,^32^ but not to the extent observed in the shuffled protein. This, in turn, has important implications for protein engineering, suggesting an untapped pool of enzyme scaffolds among archaeal enzymes from which to develop novel enzymes having extreme properties (in particular thermostability) for biocatalytic purposes.

## Results and Discussion

### Random sequence shuffling of thermophilic archaeal PTPs

**Figure 3.**
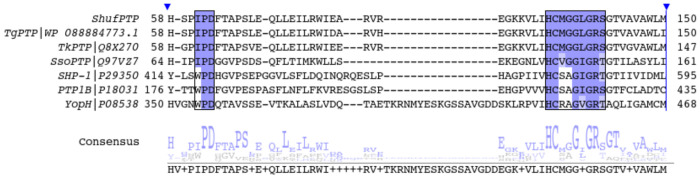
Sequence conservation demonstrates the archaeal nature of ShufPTP. Multiple sequence alignment of characterized PTPs with the synthetic PTP ShufPTP highlights its similarities to thermophilic archaeal PTPs. Shown here is a portion of the sequence alignment among two archaeal PTPs from *Thermococci*, TgPTP and TkPTP,^33^ a third archaeal PTP SsoPTP,^32^ the human PTPs SHP-1 and PTP1B, and the bacterial PTP YopH, as well as the corresponding consensus sequence. Sequence alignment was performed using T-Coffee.^50^ The conserved IPD- or WPD-regions of the acid loop and the phosphate binding P-loop ((H/V)CX5R(S/T)) are highlighted in purple. Of the non-archaeal PTPs shown here, SHP-1 is an important anticancer drug target,^51^ and PTP1B and YopH are two of the most studied PTPs to date.^18, 47^ ShufPTP shows 90% sequence identity to TgPTP, 85% to TkPTP, 39% to SsoPTP, 25% to SHP-1, 30% to PTP1B and 31% to YopH, respectively.

ShufPTP was obtained as a product of random sequence shuffling of five thermophilic archaeal PTPs (see Figure S1 for sequences and organisms used). Figure 3 shows sequence alignment between ShufPTP, three archaeal PTPs (TgPTP, TkPTP and SsoPTP, the first two of which were used for sequence shuffling), and the human and bacterial PTPs PTP1B and YopH, which are two of the best-studied PTPs,^18, 47^ for comparison.

Unsurprisingly, ShufPTP shows highest sequence similarity to the PTPs from *Thermococci*, the currently uncharacterized TgPTP, and TkPTP^33^ (90% and 85% sequence identity, respectively, see Figure 3, with E-values of 8e-101 and 2e-91, sequence alignment was performed using the Standard Protein BLAST (BLASTp) webserver^48, 49^). This high sequence similarity is to be expected given both PTPs were used for sequence shuffling, and notably, as shown in Figure 3, the P-loops ((H/V)CX_5_R(S/T)) of these three PTPs are identical, the acid-loops (IPD-loop) of TgPTP and ShufPTP are identical, and the acid-loops of TkPTP and ShufPTP differ only in one position (where a serine in ShufPTP presents as a glycine in TkPTP, likely increasing the flexibility of the TkPTP IPD-loop).

ShufPTP shares much lower sequence identity with SsoPTP (39%, with the E-value of 1e-28), although SsoPTP is also archaeal in origin.^32^ Notably, the SsoPTP IPD-loop contains two additional glycine residues, present immediately after the conserved IPD-motif of the acid-loop, which present as phenylalanine and tyrosine in ShufPTP. This again suggests that SsoPTP should have a more flexible IPD-loop than ShufPTP. Interestingly, the sequence identity between ShufPTP and SsoPTP is not much higher than between ShufPTP and the human and bacterial PTPs, SHP-1, PTP1B and YopH (25%, 30% and 31%, respectively). Based on the corresponding E-values (1e-6, 6 e-8 and 6e-4 for SHP-1, PTP1B and YopH, respectively).

Several sequence features of the archaeal PTPs TkPTP and SsoPTP have been associated with unconventional conformational dynamics,^32, 33^ some of which are present in ShufPTP as well. For instance, the P-loops of all four archaeal PTPs shown in Figure 3, including ShufPTP, have additional glycine residues compared to SHP-1, PTP1B and YopH, a GG motif which could contribute to the unusual P-loop conformational flexibility that has been observed in TkPTP and SsoPTP.^32, 33^ Mutational experiments on TkPTP have suggested that the GG motif is important (but not sufficient) for P-loop flexibility, but that the GG-motif does not affect turnover.^33^ Further, while many PTPs have a WPD sequence in their acid loop, the archaeal PTPs shown in Figure 3 carry instead an IPD sequence, which likely contributes to rigidifying the IPD-loop compared to the acid-loops of PTPs such as PTP1B and YopH (available crystal structures of SsoPTP and TkPTP, PDB IDs: 7MPD,^32^ 2I6I,^52^ 2I6P^52^ and 5Z5A,^33^ 5Z59,^33^ all show the IPD-loop in its catalytically closed conformation).

Finally, both SsoPTP, and the archaeal PTPs upon which ShufPTP was modeled (Figure S1), are thermophiles.^32, 39^ In this context, hydrophobic environment has been identified as a key factor driving the thermal stability of thermophilic proteins.^53–56^ Taking this into account, we calculated the hydrophobic content of each of the six PTPs shown in Figure 3 using the Kyte-Doolittle scale.^57^ The hydrophobic content of the human PTPs, SHP-1 and PTP1B are 34.8 and 35.9%, respectively, and that of the mesophilic bacterial PTP YopH is 35.5%. The three archaeal PTPs, SsoPTP, TkPTP and TgPTP show hydrophobic content ranging from 41% (SsoPTP) to 44% (TgPTP). Both TkPTP and SsoPTP have *T*_m_ and activity at temperatures 65 °C or above. TgPTP has not been characterized, but the source organism, *T. grogonarius*, is an extremely thermophilic archaeon that has been identified in New Zealand submarine hot vents, with optimal growth conditions at 80 °C.^58^ Assuming this link between hydrophobic content and thermostability, at 44.7%, ShufPTP has a similar hydrophobic content to TgPTP, and thus, based on the observed trends, we would expect ShufPTP to have thermal stability and activity that at least matches or even surpasses that of the three archaeal PTPs shown in Figure 3. To verify this, we have subjected ShufPTP to detailed structural, biochemical, biophysical, and computational characterization, as described in subsequent sections.

### Structural Characterization

Four crystal structures of ShufPTP were determined ranging from 1.5 to 2.1 Å resolution (Table S1). In each case, ShufPTP adopts a canonical architecture characteristic of the protein tyrosine phosphatase family, with particularly close structural similarity to its archaeal relatives TkPTP (RMSD 1.1 Å) and SsoPTP (RMSD 2.4 Å). The enzyme contains all four signature catalytic motifs that define modern PTPs: the phosphate-binding P-loop with conserved (H/V)CX_5_R(S/T) motif, the Q-loop that carries the water-directing glutamine, the IPD-loop that carries the conserved general acid (an aspartic acid), and the E-loop that carries a possible alternate general acid candidate (a glutamic acid).^59, 60^

**Figure 4.**
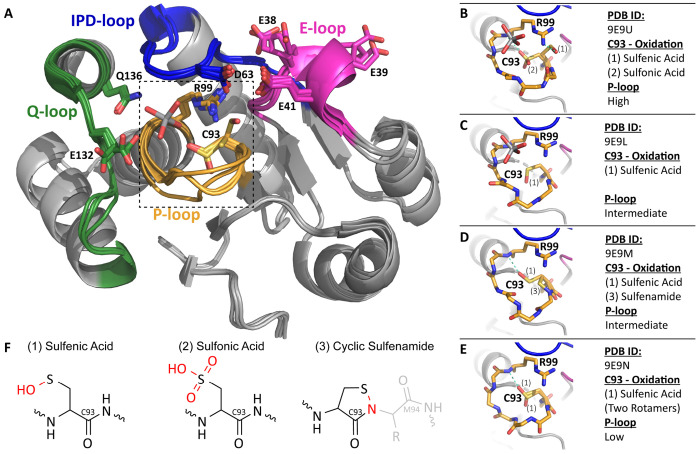
ShufPTP adopts a canonical PTP structure. (**A**) The active site of ShufPTP consists of four loop motifs, P-loop (Yellow), IPD-loop (blue), Q-loop (green), and E-loop (pink). All solved structures are largely superimposable with key differences seen in the P-loop motif. The vanadate ligand from PDB ID: 9E9U (this work) and key residues are shown in sticks. The dashed line refers to the P-loop region depicted in higher resolution in panels B-E. (**B-E**) Stick view of the different P-loop conformations and oxidation states. H-bonds are shown in teal, with differences in observed oxidation state, ligand, and conformation are listed to the right. (**F**) Chemical structure of the three observed oxidation states of C93. Atoms and bonds shown in red highlight the newly formed bonds caused by oxidation of the thiol.

In each of the ShufPTP structures, the Q-loop, IPD-loop, and E-loop conformations are largely superimposable, except for sidechain rotamer differences in E132 in the Q-loop and E38 and E39 of the E-loop (Figure 4). In each structure, the conventional general acid D63 on the IPD-loop adopts what appears to be a catalytically unproductive conformation, with its side chain orientated away from the P-loop, directed towards E41 on the E-loop. In structures in which the transition state analog vanadate was bound, it is coordinated by E132 and Q136 on the Q-loop. The interaction with Q136 is not typically observed in other vanadate-bound PTP structures and appears to be facilitated by an upward shift in the vanadate position relative to other PTP structures.

The most striking difference between the ShufPTP structures is in the P-loop. We observe three distinct P-loop backbone conformations, which we designate “low,” “intermediate,” or “high,” referring to the relative position of the loop to the substrate binding site within the active site (Figure 4). The low conformation is identical to the catalytically competent form previously observed in TkPTP (PDB ID: 5Z5A).^33^

Remarkably, the oxidation states of the catalytic nucleophile, C93 differ depending on the structure, often with multiple oxidation states observed in a single structure (Figure 4). Specifically, we observe the cysteine as: (1) a sulfenic acid, (2) a sulfonic acid, and (3) a rare cyclic five-membered sulfenamide ring (Figures 4B through F). This latter species arises from an intramolecular dehydration reaction between the sulfenic acid and an adjacent backbone amide from M94. In structures containing the sulfenic acid form, C93 does not form the canonical hydrogen bond to the central vanadium atom of the ligand, but instead is positioned away from the active site and in one case, forms a hydrogen bond with the conserved arginine R99 on the opposite side of the active site.

The relationship between the P-loop conformation and the oxidation state of C93 is unclear, in part because multiple oxidation states are often observed in a single structure (Figure 4). However, a general observation is that the oxidized C93 interacts with R99 in all conformational states except for the intermediate, vanadate bound conformation. C93 interacts with the sidechain of R99 only in the high conformation. In all cases, the R99 conformation is the same, regardless of P-loop conformation, ligand, or C93 interaction, suggesting that R99 could interact with vanadate in all P-loop conformations.

### Phosphatase Activities and Dual General Acid Catalysis

**Figure 5.**
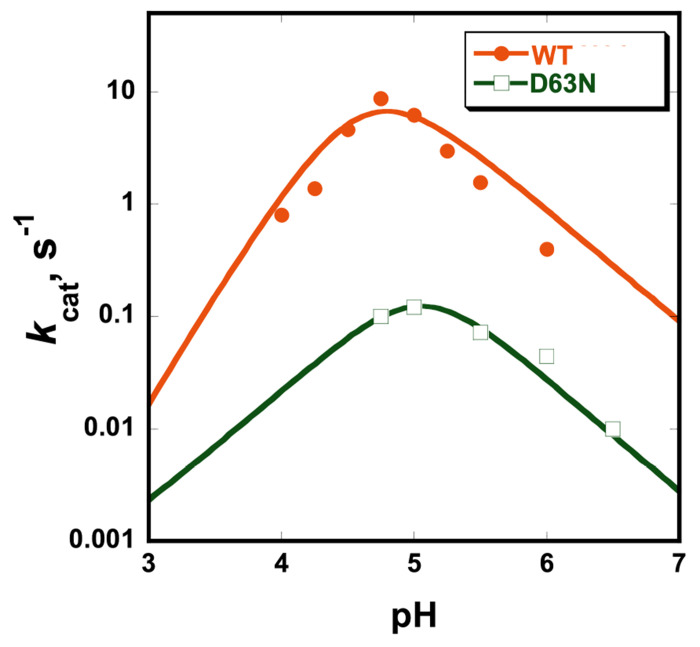
ShufPTP exhibits a bell-shaped pH-rate profile like extant PTPs. The retention of the basic limb in the profile for the general acid variant D63N indicates the presence of an alternate general acid. Precipitation of the variant below pH 4.75 precluded acquisition of data at lower pH.

ShufPTP exhibits a bell-shaped pH-rate profile like modern PTPs (Figure 5), indicating a conserved catalytic mechanism involving two essential catalytic residues, as shown in Figure 1. The optimal turnover number for ShufPTP is 8.6 ± 0.1 s^−1^ at its pH optimum 4.75 at 22 °C, approximately two-fold faster than its closest relative among the modern PTPs, TkPTP.^33^ Table 1 shows how the activity of ShufPTP compares to extant members of the PTP family.

By analogy with the structures of extant PTPs, residue Asp63 in ShufPTP occupies the position of the conventional general acid. The ShufPTP variant D63N has a turnover number of 0.1 s^−1^ at pH 5.0, approximately 10-fold faster than the corresponding variant of TkPTP , which is also D63N^33^ (Table 1). The variant exhibited precipitation, attributed to denaturation, below pH 4.5, limiting the collection of kinetic data on the acidic limb of the pH-rate profile. The general acid variant of ShufPTP exhibits an approximately 70-fold rate reduction compared to the wild-type enzyme, less than the 2 to 3-order of magnitude^64, 65^ slower activities that general acid mutants of PTPs typically exhibit. This observation, together with the retained basic limb of the pH-rate profile, suggests the existence of a second functional general acid, as has been observed in the case of VHZ^63^ and SsoPTP.^32^

### Thermostability

To assess thermal stability, catalysis by ShufPTP was measured from 22 – 90 °C. ShufPTP exhibits a turnover number of 26 ± 3 s^−1^ at 60 °C, which is comparable to TkPTP at the same temperature.^33^ The fastest activity for ShufPTP was observed at 90 °C, with a turnover number of 140 ± 20 s^−1^, approximately 16-fold faster than that at 22 °C. An Arrhenius plot (Figure S2) implies significant thermostability of ShufPTP. Temperature-induced denaturation and associated loss of activity would result in a downward curvature at high temperatures, which is not observed.

Enzyme activity determinations for ShufPTP within the 22-90 °C temperature range show an increase in the steady-state rate with temperature that conforms to the Arrhenius equation (Figure S2). Protein denaturation within the studied temperature range would have brought about a distinct decrease in activity with the concomitant deviation from the Arrhenius behavior, which is not observed in the experimental data. We are led to conclude, therefore, that the thermal denaturation of ShufPTP takes place at temperatures significantly above 90 °C. Verification of denaturation at temperatures above 90 °C is challenging, simply because the normal boiling point of water is 100 °C, which sets an upper limit to the temperature range that can be accessed by many experimental methodologies. This constraint is overcome when using differential scanning calorimetry (DSC) to study denaturation, because DSC experiments customarily involve the application of a moderate overpressure on the order of a couple of atmospheres. While the purpose of this procedure was originally to prevent bubble formation during the temperature scan, the most relevant consequence of the applied overpressure is to increase the boiling point of water and enable the temperature scans to terminate many degrees above 100 °C.

**Figure 6.**
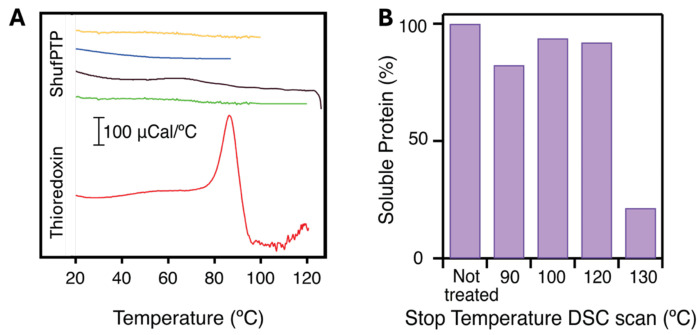
Differential scanning calorimetry (DSC) of ShufPTP. (**A**) Profiles of heat capacity versus temperature for solutions of ShufPTP and thioredoxin at 1 mg/mL and pH 7. The profiles have been shifted in the y-axis for the sake of clarity The four uppermost profiles correspond to ShufPTP solutions and differ in the upper temperature of the DSC scan, as it is visually apparent. The lowermost profile corresponds to a thioredoxin solution and, unlike the profiles for ShufPTP, shows a prominent denaturation transition (heat capacity peak). (**B**) Amount of non-aggregated (soluble) protein in solutions of ShufPTP extracted from the calorimetric cell after cooling from a DSC scan. The amount of soluble protein is given as function of the highest temperature reached in the DSC scan.

We performed DSC experiments with solutions of ShufPTP at pH 7, under an overpressure of about 2 atm. Several DSC experiments were performed terminating at different temperatures, including one terminating above 120 °C. Remarkably, none of these experiments (see upper panel in Figure 6) revealed a calorimetric transition (*i.e.*, a heat capacity “peak”) that can be attributed to protein denaturation. It is important to note that the absence of a calorimetric transition in the scans with ShufPTP cannot be attributed to instrumental limitations or to a low denaturation heat capacity signal for this protein. This is because the area under a calorimetric transition is the denaturation enthalpy, which as a first approximation, scales with the protein molecular mass.^66^ For a protein of a molecular mass of 17 kDa, such as ShufPTP, a simple calculation based on the known structure-energetics correlations (Table 5 in ref. ^66^) leads to estimates of the denaturation enthalpy of about 191, 215, 239 and 262 kcal/mol at 90 °C, 100 °C, 110 °C and 120 °C, respectively. These high denaturation enthalpies suggest that if the protein denaturation took place within the 90-125 °C temperature range, it would have been easily detected in our DSC experiments. In order to illustrate this, we performed DSC experiments with thioredoxin under the same conditions (in particular, using the same protein concentration as in the ShufPTP experiments: 1mg/mL). Thioredoxin has a molecular mass of about 11 kDa and, therefore, its denaturation enthalpy values, as estimated from the structure-energetics correlation, are substantially lower than those predicted for ShufPTP. Yet, the denaturation transition is clearly seen at about 90 °C in DSC scans with thioredoxin solutions of concentration 1 mg/mL.

Overall, the absence of a calorimetric transition in our DSC scans of ShufPTP solutions support a very high denaturation temperature for this protein. We found indirect evidence from the calorimetric experiments of the onset of denaturation at about 130°C. Thus, we detected substantial aggregation in the sample extracted from the calorimetric cell after cooling from a DSC scan in which a temperature of about 130 °C had been reached. On the other hand, aggregation was not observed in protein solutions extracted after cooling in experiments with a final temperature lower than 130 °C. For all these experiments, the protein solution extracted upon cooling was centrifuged to eliminate any aggregated protein, and the supernatant was subjected to gel electrophoresis. In all cases, a single band corresponding to the molecular weight of ShufPTP was observed, but its intensity was much smaller for the sample that had reached 130 °C in the calorimetric experiment, reflecting strong aggregation in this case (see lower panel in Figure 6). These experiments suggest that denaturation of ShufPTP occurs at temperatures of about 130 °C or above and follows a Lumry-Eyring type of mechanism,^67–69^ in which unfolding is followed by an irreversible alteration of the unfolded protein, aggregation in this case.

### Substrate Preference by Naphthyl Phosphate Isomers

The β/α-naphthyl phosphate hydrolysis ratio has been used as a reporter for the active site geometry and substrate preferences for PTPs.^59, 70–74^ The PTP superfamily includes enzymes specific for phosphorylated tyrosine residues, and the dual-specificity phosphatases (DSPs) that hydrolyze phosphorylated serine and threonine residues, as well as tyrosine. The substrate specificity of tyrosine-specific PTPs arises from a deeper and narrower catalytic site pocket compared to those of DSPs. Due to the steric and structural resemblance of α-naphthyl phosphate to phosphoserine and phosphothreonine, and β-naphthyl phosphate to phosphotyrosine, extant DSPs exhibit a β-NP/α-NP (*V/K* ratio) in the range of 1 – 2, compared to a ratio of 7 or higher for classical tyrosine specific PTPs.^75^ Thus, this assay provides insight into ShufPTP substrate preference compared to extant PTP family members. Furthermore, given the P-loop conformational changes of the similar TkPTP due to heat treatment,^33^ it was suspected that ShufPTP might exhibit similar temperature-dependent conformational changes, and thus, a change of active site geometry and substrate preference. The ratio of (*V/K*) for β- to α-naphthyl phosphate substrate consumption catalyzed by ShufPTP is 1.7 at 22 °C and 1.8 at 60 °C, indicating that ShufPTP does not exhibit temperature-dependent changes in substrate preference or active site geometry, and that its substrate preference is more analogous to extant dual-specificity phosphatases (DSPs) than tyrosine-specific PTPs.

### Enzyme Inactivation by Hydrogen Peroxide

Reversible oxidation of cysteine residues plays an essential regulatory role in enzyme catalysis and gene regulation,^76–80^ and specifically, oxidation regulates tyrosine phosphorylation.^81–84^ Several *in vitro* studies have identified hydrogen peroxide as a specific inactivator for PTPs.^40, 41, 60, 85–91^ The evidence for the cysteine sulfenic acid and sulfonamide intermediate forms support the theory for reversible cellular redox-regulated PTP activities. In order to rationalize the observed oxidation states of C93 in the X-ray crystal structures (Figure 4), we measured the second order rate constant for inactivation of ShufPTP by hydrogen peroxide and compared it to reported values for extant PTPs (Table 2). ShufPTP exhibits a higher oxidation rate than any of the previously reported values for PTPs, with a *k*_inact_ of 65 ± 5 M^−1^ s^−1^. The reasons for the higher propensity of ShufPTP toward oxidation by hydrogen peroxide remain to be determined.

### Identifying Prospective Catalytic Backup Residues

Visual examination of all available crystal structures of ShufPTP led to the identification of several carboxylate side chains located near the active site (Figure S3). While some of these residues point away from the active site, they are all located on mobile loops (D63 on the IPD-loop, E38, E39 and E41 on the E-loop, and E132 on the Q-loop), making it plausible that loop fluctuations could render these side chains transiently conformationally accessible for catalysis, allowing them to act as backups.

**Figure 7.**
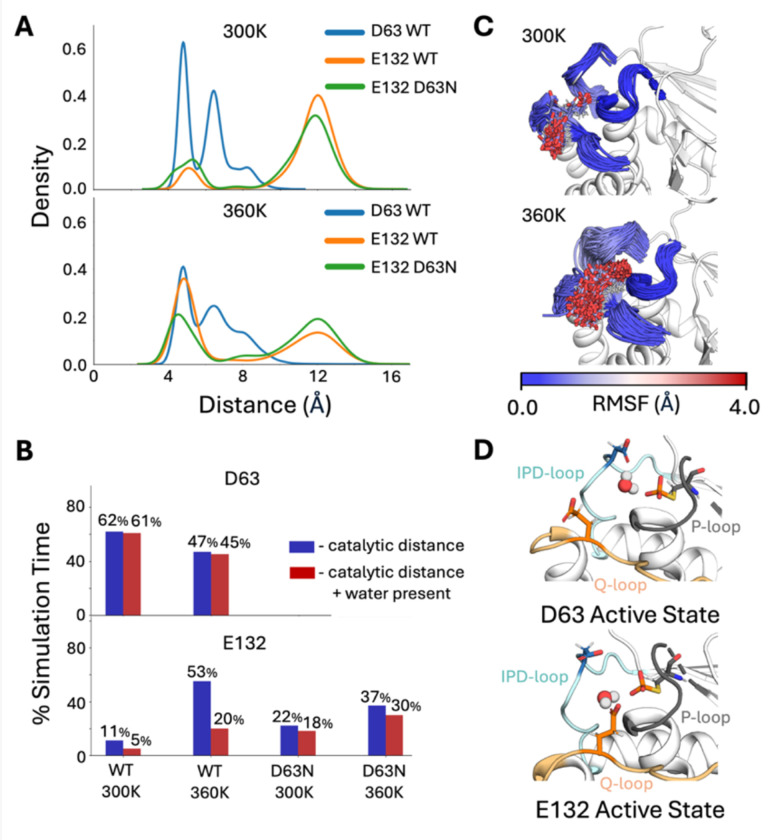
Characterizing prospective catalytic backups in ShufPTP. Potential backups identified by tracking the distances between the phosphate group at the phosphoenzyme intermediate and prospective catalytic carboxylate side chains, extracted from our MD simulations of wild-type and D63N ShufPTP. (**A**) Kernal density estimates (KDE) of the distances between the phosphate group and prospective catalytic residues, defined as the distance between the P atom of the phosphate group and the closest oxygen atom of the carboxylate side chain of each key residue. (**B**) The % simulation time the side chains of each of the three most likely candidates (D63, E41 and E132) spent within 6Å of the phosphate group phosphorus atom (shown in blue). Further, to be catalytically viable, it is necessary for a water molecule to bridge the respective side chain and the phosphate group, in order to act as a nucleophile (Figure 1). For each prospective catalytic side chain, we calculated the % of simulation time a water molecule is positioned within both 3.5Å of the carboxylic acid of the respective side chain, and of the phosphorus atom of the phosphate group (red, measured based on distances to the nucleophilic oxygen atom). (**C**) The dramatic increase in proximity of E132 to the C93 side chain with increased temperature can be attributed to the increased flexibility of the Q-loop. (**D**) Illustration of the catalytically active conformations of D63 (IPD-loop) and E132 (Q-loop), with a catalytic water molecule bridging the side chain and the phosphate group.

To validate this hypothesis, we tracked the distances between the side chains of E38, E39, D63, E41 and E132 during MD simulations of the phosphoenzyme intermediate states of low P-loop state wild-type and D63N ShufPTP (PDB:9E9N, this work), at both 300 and 360K (Figures 7, and S4). For this analysis, we focused our simulations on the phosphoenzyme intermediate state of ShufPTP, as we expect hydrolysis to be rate-limiting, based on burst kinetics of PTP1B and YopH.^20, 21^ The low structure of the P-loop was chosen for this analysis, due to its structural similarity to the catalytically active state of TkPTP.^33^ Additionally, when simulated in the absence of the phosphate, this starting structure was the only one out of four ShufPTP crystal structures that demonstrated positional stability of the side chain R99, which is a key conserved residue important for phosphate binding in the active site^92^ (Figure S5).

Based on the analysis of phosphorylated wild-type and D63N simulations, only two residues sample conformations within a plausible reactive distance of the phosphoenzyme intermediate in the wild-type enzyme: the native acid, D63, and E132, which is located on the Q-loop. The distance distribution of E132 shows two peaks: a dominant non-reactive peak, and a second, smaller peak at ~4.1 Å, which plausibly leads to a reactive conformation. We repeated this analysis at 300K in the D63N variant, and at 360K in both variants (Figures 7 and S4), demonstrating that both abolishing the native catalytic residue and increasing temperature increases sampling of prospectively catalytic conformations of E132, during the simulations of both ShufPTP and of the D63N variant.

We note that for the hydrolysis reaction to occur, it is essential to have a water molecule correctly positioned for both activation by the respective glutamic acid side chain, and for nucleophilic attack on the phosphorus group of the intermediate. To account for this, we also tracked water molecules that bridge the D63, E132, and E41 side chains and the phosphate group, and are correctly positioned for reaction (*i.e.* within 3.5Å of both the carboxylate and phosphate groups) during our simulations (Figures 7B and S4B), indicating that E132 is at least transiently aligned for catalysis during our simulations, in particular at 360K (where we see a drop in catalytic conformations of D63). Finally, as shown in Figure 7C and D, the increase in catalytic conformations of the E132 side chain at higher temperature are facilitated by increased flexibility of the Q-loop, allowing for a coordinated rearrangement of the IPD- and Q-loops relative to each other, where D63 points out of the active site, allowing E132 to enter the active site as the Q-loop moves closer to the active site. This rearrangement could facilitate the role of E132 as a backup.

### Empirical Valence Bond Simulations of Phosphoenzyme-Intermediate Hydrolysis

To verify whether our hypothesis with regards to the role of E132 as a catalytic backup is correct, we performed empirical valence bond (EVB) simulations of phosphoenzyme-intermediate hydrolysis in wild-type and D63N ShufPTP, facilitated by either D63 or E132 in each enzyme. Based on prior work on other enzymes with catalytic backups, one would expect that any backup mechanism observed in the D63N variant would also exist in the wild-type enzyme, albeit less effectively than the native mechanism,^93–95^ and this will likely also be the case for ShufPTP. Since the crystal structure of ShufPTP demonstrates only catalytically unproductive conformation of the D63 side chain (with this residue pointing outside the active site and forming what appears to be a low-barrier hydrogen bond with the side chain of E41 on the E-loop), EVB simulations were initialized from snapshots taken from our MD simulations with catalytically active Asp/Glu conformations, as described in the Materials and Methods.

**Figure 8.**
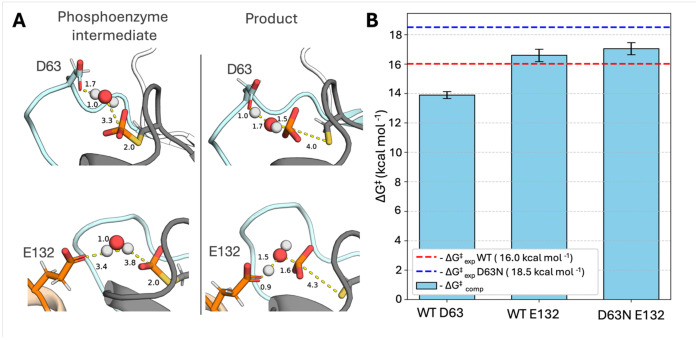
Empirical Valence Bond evaluation of reaction barrier for the dominant and backup mechanism of ShufPTP. **(A)** Representative snapshots of phosphoenzyme intermediate and product state structures extracted from empirical valence bond (EVB) simulations^96^ of the hydrolysis reaction catalyzed by wild-type ShufPTP. Shown here are stationary points for the D63-as-base and E132-as-base mechanisms. (**B**) Calculated ΔG^‡^ values (kcal mol^−1^) for the D63-as-base and E132-as-base mechanisms in wild-type (WT) and D63N ShufPTP were compared to the experimental values obtained from the kinetic data (*k*_cat_, Table 1) for both variants. The red dashed line indicates the experimental activation free energy for the reaction catalyzed by wild-type (WT) ShufPTP, and the blue dashed line indicates the experimental activation free energy for the reaction catalyzed by the D63N ShufPTP mutant. The error bars represent the standard error of the mean on the calculated activation free energies over 20 individual EVB trajectories for each system. The raw data for this figure are shown in Table S2.

We note that for our EVB simulations, as in our MD simulations, we focus on modeling the hydrolysis of the phosphoenzyme intermediate rather than nucleophilic attack on the Michaelis complex (Figure 1), as hydrolysis of this intermediate is expected to be the rate limiting step of catalysis.^20, 21^ Beyond investigating the impact of the choice of the residue that facilitates the reaction, we assessed whether the complementary proximity of D63 and E132 to the active site has a noticeable effect on the activation barrier of the reaction (*i.e.* whether both or only one of these side chains points into the active site simultaneously). Therefore, to represent a complete set of plausible electronic environments, we considered the five following catalytic scenarios: (1, 2) wild-type ShufPTP system with D63 as the catalytic residue and E132 pointing in or out of the active site, (3, 4) wild-type ShufPTP with E132 as the catalytic residue and D63 pointing in or out of the active site, and (5) the D63N ShufPTP mutant, in which the reaction is facilitated solely by E132 (Figures 8 and S6, and Table S2).

Our EVB simulations (performed at 300K) indicate that the energetically preferred active site conformation for ShufPTP is one where both the D63 and E132 side chains point into the active site, as the electrostatic repulsion between these two side chains facilitates easier proton abstraction from the nucleophilic water molecule (Figure 1). In wild-type ShufPTP, in all scenarios, proton abstraction in a D63-as-base mechanism has a lower activation free energy than in an E132-as-base mechanism. However, the difference in energy between the two mechanisms in the preferred conformation (both side chains pointing into the active site) is only 2.7 kcal mol^−1^, with a calculated activation free energy of 13.9 kcal mol^−1^ for the D63-as-base mechanism, and a calculated activation free energy of 16.6 kcal mol^−1^ for the E132-as-base mechanism. For reference, the corresponding experimental value is 16.6 kcal mol^−1^ (Table S2), based on a *k*_cat_ of 8.6 s^−1^ (Table 1), calculated using transition state theory. Thus, both plausible mechanisms are energetically within range of the corresponding experimental activation free energy, with a preference for the native D63-as-base mechanism.

Interestingly, the calculated energy difference between the D63- and E132-as-base mechanisms (2.7 kcal mol^−1^) is very close to the difference between the experimental activation free energies for the reactions catalyzed by wild-type and D63N ShufPTP (2.5 kcal mol^−1^, Tables 1 and S2). Indeed, our EVB simulations of the E132-as-base mechanism in D63N ShufPTP yield an activation free energy of 17.1 kcal mol^−1^, which is slightly lower than the experimental value (18.5 kcal mol-1), but one should also take into account that E132, which is also on a mobile loop, samples a catalytic conformation less frequently than D63 on the WPD-loop (Figure 7).

The calculated activation free energies for E132-as-base mechanism in wild-type ShufPTP, obtained from starting conformations in which D63 points out of the active site (17.4 kcal mol^−1^, Table S2), are very similar to the calculated activation free energy for the D63N mutant (17.1 kcal mol^−1^). This suggests that (1) both D63- and E132-as-base mechanisms are catalytically plausible in wild-type ShufPTP, with D63 dominating due to both a lower activation free energy and more frequent sampling of catalytic conformations, and (2) at least part of the loss of activity in the D63N mutant is not due to the loss of catalytically favorable ground state destabilization, but rather due to elimination of the charge on the D63 side chain from the active site, making the E132-as-base mechanism about 0.4 kcal mol^−1^ (from EVB calculations) energetically less favorable than in wild-type ShufPTP.

### Simulations of IPD- and P-Loop Dynamics in ShufPTP and TkPTP

Class I PTPs are characterized by the motion of the flexible loops decorating their active sites, with a mobile WPD-loop, that undergoes significant conformational transitions during catalysis (Figure 1), and a rigid phosphate binding P-loop.^47^ In contrast, the analogous acid loop (the IPD-loop) in archaeal PTPs, including ShufPTP, appears to prefer to be in a closed or semi-closed conformation (see PDB IDs: 5Z59,^33^ 5Z5A^33^ and 7MPD^32^), while the P-loop is conformationally flexible^32, 33^ (Figure 2). Further, in SsoPTP, even the Q-loop undergoes conformational exchange motions, as evidenced by NMR spectroscopy.^32^ As ShufPTP shares 90% sequence identity with the extant archaeal PTP, TkPTP, we have performed a comparative analysis of IPD- and P-loop flexibility in these two enzymes based on our molecular dynamics simulations. In particular, despite their high sequence identity, the increase in thermal stability and catalytic efficiency of ShufPTP, compared to TkPTP,^33^ suggests putative differences between the conformational dynamics of these two enzymes. We thus also compare the conformational dynamics of key catalytic loops in wild-type and D63N ShufPTP at both 300K and 360K, to understand both baseline dynamics and how they are affected by temperature.

**Figure 9.**
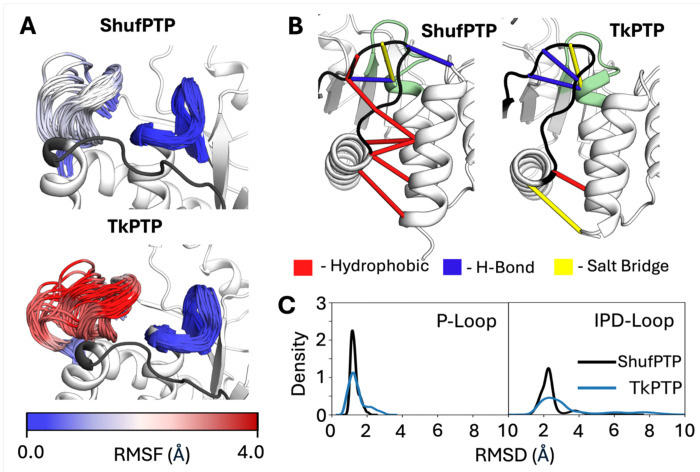
Conformational dynamics of the IPD- and P-loops in the unliganded forms of ShufPTP and TkPTP. Simulations were initiated from the IPD-loop-closed and low P-loop state of each enzyme. (**A**) The ensemble of sampled conformations during simulations of ShufPTP and TkPTP at the IPD-loop-closed unliganded state was visualized and colored based on the root mean square fluctuations (RMSF, Å) of loop C_α_-atoms. (**B**) A stabilizing hydrophobic network at the base of the IPD-loop of was identified and compared to the interactions present in TkPTP. This network was obtained by calculations using Key Interaction Networks (KIN).^97^ (**C**) Assessment of the P-loop and IPD-loop conformational ensembles based on kernel density estimation (KDE) analysis of the root mean square deviations (RMSD, Å) of the backbone atoms of the IPD- and P-loops of ShufPTP and TkPTP. These data indicate a less conformationally diverse ensemble in TkPTP than in ShufPTP.

Figures 9 and S7 show comparisons of IPD- and P-loop dynamics and conformational distributions in ShufPTP and in TkPTP, starting from the unphosphorylated enzyme and the phosphoenzyme intermediate, respectively, with the IPD-loop in a closed conformation at the start of the simulation. In the case of the unliganded simulations, we observe that the TkPTP IPD-loop is conformationally flexible (similar to dynamics observed in PTP1B in our prior simulations^16^). In contrast, and in agreement with our crystal structures of ShufPTP (in which the IPD-loop is primarily in a closed conformation, PDB IDs: 9E9N, 9E9L, 9E9M, 9E9U), the conformational ensemble of the IPD-loop (and even the P-loop), is less diverse and more restricted, and mostly concentrated on sampling catalytically optimal conformations associated with positioning D63 closer to the active site, while also stabilizing C93.

The restricted loop dynamics in ShufPTP can be partially rationalized by the extended C-terminal helix of the IPD-loop of ShufPTP, which carries additional hydrophobic residues such as F146, which is not present in TkPTP. This, in combination with a bulkier leucine at position 69 (rather than valine in TkPTP) reinforces a network of hydrophobic interactions that likely limit the opening of the IPD-loop and fix the conformation of this loop in its catalytically active closed conformation (Figure 9B). Further, our simulations at the phosphoenzyme intermediate state indicate that the P- and IPD-loop are rigidified in the presence of phosphoenzyme intermediate, in what is likely a ligand-gated conformational change, as observed in our prior work on PTP1B and YopH.^16^ Figure S7 shows that, in agreement with prior work,^33^ conformational dynamics of the P- and IPD-loops of TkPTP and ShufPTP exhibit temperature sensitivity. While both unliganded systems show broader conformational distributions at 360K than 300K, at the phosphoenzyme intermediate state, TkPTP exhibits a narrowing of its loop conformational ensemble with increasing temperature, while the corresponding conformational dynamics of ShufPTP remains largely unaffected.

We further observe that both TkPTP^33^ and ShufPTP show unusual conformational flexibility in their phosphate binding P-loops (Figure 2), which is particularly curious given that phosphate-binding P-loops tend to be rigid^16, 28–32, 98^ and fall within clearly defined ranges of conformational parameters (see *e.g.*, ref. ^98^ for characterization of the analogous Walker A P-loop). In particular, X-ray crystallographic structures of both TkPTP and ShufPTP show both catalytically active and inactive conformations of the phosphate-binding P-loops (Figure 9C), that differ by 0.4Å RMSD between their backbone atoms. In order to characterize transitions between these states in our MD simulations, the following root mean square deviation (RMSD)-like metric was used to compute the similarity between simulation frames extracted from our MD simulations, and the corresponding active/inactive conformations in the X-ray crystallographic structure (CS):

(1) $$Angle\;RMSD(MD,CS)=\sum_{i}^{P-loopresidues}\frac{1}{N}{\left(\Phi_{i}-\phi_{i}\right)}^{2}+{\left(\Psi_{i}-\psi_{i}\right)}^{2}$$

Here, *N* is the total number of P-loop residues considered, $\left(\Phi_{i},\Psi_{i}\right)$ are backbone angles of the residue *i* in the reference crystal structure and $\left(\Phi_{i},\Psi_{i}\right)$ are corresponding angles from the simulation frame. This analysis was then applied to simulations of wild-type ShufPTP and TkPTP initiated from active (low), intermediate and inactive (high) conformations of the P-loop, with the IPD-loop in its loop-closed conformation, in agreement with existing structural data (ShufPTP PDB IDs: 9E9N, 9E9L, 9E9M and 9E9U, this work, and TkPTP PDB IDs: 5Z59 and 5Z5A^33^).

**Figure 10.**
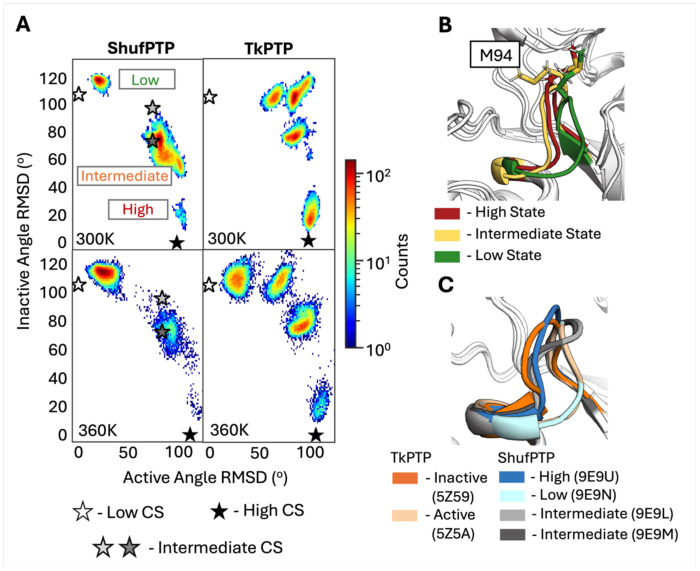
Comparison of P-loop conformational transitions between ShufPTP and TkPTP. (**A**) 2D histograms of the P-loop conformation sampled in our MD simulations of each enzyme, defined based on a root mean square deviation (RMSD)-like metric (Eq. 1 of the main text). The deviation between active (low) and inactive (high) P-loop state X-ray crystal structures (CS) of ShufPTP and TkPTP corresponds to φ and ψ angles of 116.1 degrees and 104.7 degrees respectively. The angle RMSD of two ShufPTP intermediates to active and inactive CS are: 9E9L = (78.6, 95.2) degrees, and 9E9M = (78.6, 71.6) degrees. Simulations were initiated from inactive (high) P-loop states of each enzyme, with the IPD-loop in its loop-closed state. Corresponding analysis initiated from the intermediates and active (low) P-loop state is shown in Figures S8 and S9. The corresponding crystallographic positions of the P-loop are denoted by stars, as annotated on the figure. (**B**) Illustration of structures from the local maxima of the three states observed on the histogram of ShufPTP, in order to illustrate the key structural differences between the MD intermediate and the inactive (high) state highlighted. (**C**) Comparison of active (low), intermediate and inactive (high) P-loop conformations of TkPTP and ShufPTP, based on crystallographic data (PDB IDs: 5Z5A,^33^ 5Z59,^33^ 9E9N, 9E9L, 9E9M and 9E9U).

When simulations are initialized from the active (low) P-loop state, both ShufPTP and TkPTP sample highly stable localized conformations of the P-loop, which aligns with the catalytic importance of this conformation (Figure S8). Figure 10 illustrates that, in comparison to TkPTP, the inactive (high) state of the ShufPTP P-loop is severely destabilized and demonstrates a full transition towards an active (low) state at 300K. Even in the 360K simulations ShufPTP samples fewer intermediate conformations and demonstrates a rapid transition towards an active state. Specifically, the ShufPTP P-loop rapidly adopts an intermediate conformation between the two states, where the M94 side chain is rotated towards the active site of this PTP, further driving the transition towards the low P-loop state (Figure 10B). This transitional state observed in MD is in good agreement with crystalized intermediates, particularly the intermediate without vanadate at the active site (PDB ID: 9E9M) (Figure S8). In contrast, TkPTP explores a variety of intermediate conformations during our simulations, but the active conformation is reached only in the conditions of elevated temperature. The fact that we observe these transitions in ShufPTP in the 300K simulations but not in TkPTP already on the 500ns MD simulation timescales suggests a lower thermodynamic barrier between the inactive and active conformation of the ShufPTP P-loop, which would (1) partially explain its increased catalytic activity at room temperature (Table 1), and (2) explain why the TkPTP P-loop transition appears to be temperature dependent,^33^ in order to surmount this barrier. These differences are particularly intriguing, given the fact that ShufPTP and TkPTP have identical P-loop sequences (Figure 3).

## Overview and Conclusions

Protein tyrosine phosphatases have proven to be an excellent model system for understanding the links between loop dynamics, catalysis, and enzyme evolution. These enzymes are regulated by the motion of a catalytic acid-loop, that carries an evolutionarily conserved aspartic acid important for acid-base catalysis in PTPs.^47^ Structural, NMR and molecular simulation studies have emphasized the links between loop motion and catalysis in these enzymes.^16, 22, 27, 47, 99^

While PTPs as a family have been studied extensively,^47, 100, 101^ archaeal PTPs have been much less focused on, and of those that have been studied, trends demonstrate unusual biochemical and biophysical properties, including hyper-thermostability, catalytic backups, and mobile phosphate binding loops.^32, 33, 102^ A better understanding of archaeal PTPs thus plays a dual role both in expanding our understanding of loop dynamics and catalysis in PTPs and loopy enzymes more broadly, but also of how enzymes evolve to operate under inhospitable conditions, given that archaea are extremophiles.^103^ In this work, we have generated an extremophile chimeric PTP, based on amino acid shuffling of five hyper-thermophilic archaeal PTP sequences (Figure 3), which we denote ShufPTP, and extensively biochemically, biophysically, structurally, and computationally characterized this enzyme.

Archaeal enzymes are known to have unique structure-function properties,^104–106^ and ShufPTP is no exception. Dynamically and in terms of stability, calorimetry indicates that ShufPTP is extremely thermostable, with denaturation likely occurring at temperatures > 130 °C (Figure 6). This is particularly noteworthy, given that the highest recorded temperature for the growth of an archaeal strain, from *Methanopyrus kandleri*, is 122 °C.^103^ Further, ShufPTP has a rather rigid acid loop (Figures 4 and 9), in contrast to the mobile acid loops observed in other PTPs.^47^ In contrast, the phosphate binding loop of ShufPTP is highly mobile, taking on at least three distinct stable conformations (Figures 4 and 10), a feature that is highly uncommon in P-loop containing enzymes more broadly, given that phosphate binding P-loops tend to be structurally rigid and follow narrowly defined geometric parameters.^16, 28–32, 98^

Mechanistically, the active site cysteine of ShufPTP is readily amenable to oxidation (Figure 4), a feature that has emerged to be an important regulatory mechanism that links cellular tyrosine phosphorylation with signaling by reactive oxygen or nitrogen species.^44^ ShufPTP is also catalytically versatile, utilizing a backup mechanism involving a glutamic acid on a mobile loop as a redundancy for its preferred Asp-as-base mechanism (utilizing the aspartic acid on the acid-loop, Figures 7 and 8). Such backup mechanisms have been seen before in an organophosphate hydrolase^93, 94^ and in quorum-quenching lactonases,^95^ but these are scavenger enzymes that are likely to be functionally diverse, and not just substrate/catalytically but also mechanistically promiscuous. Here, we show clear mechanistic promiscuity in an enzyme from a family of enzymes that is involved in regulation of core cellular processes,^47^ where fine-tuning activity and selectivity is crucial. We demonstrate through simulations that tight regulation of the relative mobility of the loops decorating the active site facilitates interchanging catalytic mechanisms.

Each of these features alone would render ShufPTP a highly unusual enzyme. Taken together, it is a perfect illustration of the extreme properties observed in archaeal enzymes more broadly.^104–106^ Importantly, ShufPTP is highly similar to extant PTPs, and shares 85% sequence identity with TkPTP and 90% sequence identity with TgPTP, two of the sequences used in the sequence shuffling. Despite this high similarity to extant PTPs, ShufPTP shows much more extreme biochemical and biophysical properties than either of the previously characterized archaeal PTPs, TkPTP^33^ and SsoPTP,^32^ to which ShufPTP shares 85 and 39% sequence identity, respectively. These data showcase how a simple jump in evolutionary space can radically alter an enzyme, while maintaining high levels of the native activity, and simultaneously greatly diversifying the enzyme’s biophysical profile. This further underscores the potential of engineered archaeal enzymes in biotechnology.^104, 105, 107–110^

## Materials and Methods

### Chemicals

Dithiothreitol (DTT) and ampicillin (AMP) were purchased from GoldBio. Protease-inhibitor tablets were purchased from Sigma-Aldrich. All other buffers and reagents were purchased from Sigma-Aldrich or Fisher. The substrate p-nitrophenyl phosphate (*p*NPP) was synthesized using published methods.^111^ Crystallography screens, trays, and coverslips were purchased from Hampton Research.

### Protein Expression and Purification

The plasmid pET-21+ encoding ShufPTP and its variant D63N were synthesized by Twist Bioscience (San Francisco, CA). The DNA was transformed into *Escherichia coli* BL21-DE3 cells and grown overnight at 37 °C on Luria-Bertani (LB) culture plates containing 100 μg mL^−1^ ampicillin. One colony was selected and placed into 10 mL of SOC media containing 100 μg mL^−1^ ampicillin and grown overnight. The following morning, 1 L of LB media containing 100 μg mL^−1^ ampicillin was inoculated with the 10 mL of overnight growth and shaken at 170 rpm at 37 °C until the optical density at 600 nm (OD600) reached 0.6–0.8. After the optimal OD was reached, the 1 L growth was induced by 0.1 mM isopropyl β-D-thiogalactoside (IPTG) and shaken at 170 rpm at room temperature overnight. The cells were harvested by centrifugation at 12,000g for 30 min at 4 °C and stored at −80 °C.

ShufPTP cells were thawed on ice and resuspended in 10x their equivalent volume of lysis buffer (50 mM imidazole pH 7.5, 1 mM ethylenediaminetetraacetic acid (EDTA), 7 mM dithio-threitol (DTT), 5 mM tris(2-carboxyethyl)phosphine (TCEP), and 10% glycerol) supplemented with protease inhibitors (0.5 mg mL^−1^ aprotinin, 0.7 mg mL^−1^ pepstatin, and 0.5 mg mL^−1^ leupeptin). The cells were lysed by sonication. The cell lysate was centrifuged at 29,000g for 30 min at 4 °C. The supernatant was heated to 70 °C for 15 minutes and filtered with a 0.45 μm syringe filter.

The filtrate was purified using a 5 mL HiTrap Q HP column equilibrated with lysis buffer on a fast protein liquid chromatography (FPLC) system. The cell lysate was loaded at 1.5 mL min^−1^ and washed with lysis buffer until the absorbance at 280 nm reached baselined. Flow-through fractions exhibiting absorbance at 280 nm were collected and tested with para-nitrophenyl phosphate (pNPP) for phosphatase activity. Active fractions were analyzed for purity using SDS-PAGE on 20% gels.

The active fractions were pooled (30-40 mL), concentrated to less than 12 mL, and loaded onto a pre-equilibrated HiLoad 26/60 Superdex 200 prepgrade column (GE) equilibrated with a buffer containing 30 mM Tris buffer pH 7.5 containing 25 mM sodium chloride, 0.2 mM EDTA, 7 mM DTT, and 5 mM TCEP. Fractions were assayed with pNPP for activity and analyzed for purity using 20% SDS-PAGE. Pure protein was concentrated to 5–10 mg mL^−1^ and either used immediately for crystallization or supplemented with 10% glycerol, flash-frozen in liquid nitrogen, and stored at −80 °C in aliquots.

### Crystallization and Structure Determination

Crystals for ShufPTP were grown by hanging drop vapor diffusion using 8 mg mL^−1^ protein and a precipitant solution of 0.1 M CAPS pH 10.5, and 30% PEG 400 at a 1:1:0.2 protein : well : 20% additive screen drop ratio. The additive screen contained 0.2 M ammonium sulfate ((NH_4_)_2_SO_4_), 20-30% polyethylene glycol 4000 (PEG 4000), and 10-35% glycerol. The vanadate bound structure was obtained by adding 5 mM sodium metavanadate (Na_3_VO_4_) to the protein for co-crystallization. Crystals were transferred to a cryo-protectant solution containing mother liquor, 20% additive screen, and 10-15% glycerol before flash freezing in liquid nitrogen.

Diffraction data were collected on the Stanford Synchrotron Radiation Lightsource (SSRL) beamline 9-2 or on a home source. Data were indexed and processed using DENZO and SCALEPACK in the HKL3000 program suite.^112^ Molecular replacement was performed with Phaser-MR as implemented in Phenix using wild-type TkPTP (PDB ID: 5Z5A) as a search model. Phenix.refine (version 1.21.2-5419)^113^ was used for refinement. Model building was performed using Coot.^114^ All figures of the enzyme structures and structural alignments therein were made using PyMOL.^115^

### Differential Scanning Calorimetry (DSC)

DSC experiments were carried out using a VP-DSC calorimeter from MicroCal, following protocols we have described previously in detail.^116^ Briefly, protein solutions for the calorimetric experiments were prepared at a concentration of 1 mg/mL in 50 mM sodium acetate, 100 mM Tris, 100 mM Bis-Tris pH 7. The same buffer was used to fill the reference cell of the calorimeter. All DSC scans were carried out under an overpressure of about 2 atm and at a scanning rate of 1.5 degrees per minute. Before each experiment with protein solution in the sample cell, several buffer-buffer baselines were recorded to ensure calorimeter equilibration.

### Steady-State Kinetics

Steady-state kinetic parameters were measured at temperatures from 22 to 90 °C. For the pH-rate profiles, concentrated aliquots of ShufPTP and its variant D63N were thawed on ice and diluted with a buffer base mix (BBM) containing 50 mM sodium acetate, 100 mM Tris, and 100 mM bis-Tris from pH 4.0 to pH 6.25. This buffer system maintains constant ionic strength throughout the pH range examined. For the Arrhenius plot, aliquots of ShufPTP were diluted with the same buffer mix at the pH optimum of 4.75. At this pH the primary buffering component is acetate, which shows a minimal temperature effect on its p*K*_a_.

A 50 mM solution of the disodium salt of *p*NPP was prepared in the same buffer mix. The reactions were run on 96-well plates using diluted enzyme and a range of substrate concentrations. Reactions were allowed to proceed for 5 min and quenched using 50 μL of 5 M NaOH, and the amount of the product *p*-nitrophenol was assayed from the absorption at 400 nm using the molar extinction coefficient of 18,300 M^−1^ cm^−1^. Reaction blanks were made using identical conditions, replacing the enzyme with buffer to correct for non-enzymatic hydrolysis of the substrate. The amount of product released and elapsed time were used to calculate the initial rates. The data were fitted to the Michaelis–Menten equation to obtain the kinetic parameters. Kinetic data were obtained on both ShufPTP and it variant D63N as a function of pH to obtain pH-rate profiles. The bell-shaped pH-rate profiles were fitted to Eq 1, the standard equation relating the dependence of the observed k_cat_ to the maximal, or limiting, value as a function of pH, where catalysis is dependent on two ionizable residues, one protonated and the other deprotonated.^117^

(1) $$k_{cat}=\frac{k_{cat}^{lim}}{\left(1+\frac{\left[H^{+}\right]}{K_{E1}}+\frac{K_{E2}}{\left[H^{+}\right]}\right)}$$

### Active-Site Cysteine Inactivation using Hydrogen Peroxide

Enzyme stock was diluted in BBM at pH 4.75 with 0.1-0.8 mM hydrogen peroxide. Reaction blanks were made with the same reagents omitting the hydrogen peroxide. The enzyme was incubated with peroxide, and aliquots were removed to assay for activity with 7.6 mM *p*NPP. Phosphatase activity assays proceeded for 5 minutes in BBM at pH 4.75 and quenched with 5 M NaOH. The formation of *p*-nitrophenol product was assayed as described above.

At each hydrogen peroxide concentration, residual activities as a function of time were plotted to obtain *k*_obs_ (s^−1^) for the inactivation rate constant at hydrogen peroxide concentration. These *k*_obs_ values were plotted against hydrogen peroxide concentrations to obtain the second-order rate constant *k*_inact_ (M^−1^ s^−1^). The same methodology was used to obtain the *k*_inact_ for YopH and PTP1B.^60^

### Substrate Preference Determination using an NMR-based competitive method

These experiments and NMR analyses were conducted following our recently described methodology.^118^ A substrate mixture containing 5 mM of both α- and β-naphthyl phosphate were made in BBM pH 5.0. Phosphatase enzymes were diluted to 0.7 μM in BBM and allowed to react with both naphthyl phosphate substrates for 20 minutes to reach a fraction of reaction of approximately 20%, and the reactions were quenched with 50 μL 1 M NaOH. 500 μL of the quenched reaction mixture was added to an NMR tube, with a stem coaxial insert tube containing phenyl phosphonic acid at a concentration of 50 mM in D_2_O. The operational frequency for ^31^P collection was 202.46 MHz and ran at 296.2 K. ^31^P 90° pulse widths were measured using a pulse delay of 60 seconds and inverse gated decoupling (Bruker pulse sequence: zgig) to avoid NOE effects. ^31^P T1 measurements were performed using the optimized 90° pulse and inverse gated decoupling (bruker pulse sequence: t1irig). Chemical shifts were calibrated by external standard (H_3_PO_4_) using a co-axial tube to ensure buffer integrity.

Kinetic mixture samples were run with 32 scans, a relaxation delay of 35 seconds, an offset frequency of 6ppm, a spectral width of 46 ppm, and a 32k points collection. Processing was performed on the software package MestReNova 14.2. The data was zero-filled to 64k points, and a line broadening factor of 1 Hz was applied. Peak areas were measured using quantitative global spectra deconvolution with 4 fitting cycles.

### Molecular Dynamics Simulations

Molecular dynamics (MD) simulations were performed to simulate the dynamical behavior of TkPTP, ShufPTP, and the ShufPTP D63N variant, considering both the active (PDB ID: 5Z5A^33^ and 9E9U) and inactive (PDB ID: 5Z59^33^ and 9E9N) forms of the P-loop. Additionally unphosphorylated intermediate forms of ShufPTP (PDB ID:9E9L, 9E9M) were simulated to obtain a comprehensive description of P-loop dynamics. The IPD-loop is in its closed conformation in all available crystal structures. Simulations of TkPTP and ShufPTP were initiated from their respective crystal structures; in the absence of crystallographic data, D63N ShufPTP was constructed using the PyMOL^115^ Mutagenesis Wizard (selecting the Asn rotamer most similarly located to the catalytic Asp in the TkPTP structure).

Simulations were performed both in the unliganded and phosphoenzyme intermediate states of structures with an active (low) P-loop conformation, and in only the unliganded state of structures with inactive (high) and intermediate conformation of their P-loop (as this conformation does not accommodate the phosphoenzyme intermediate). We note that the cysteine in the ShufPTP crystal structures is in an oxidized form; to mimic a catalytically active state of this enzyme, the C93 side chain was converted back to its reduced form using the PyMOL^115^ Mutagenesis Wizard. As two rotamers of crystalized C93 were observed in the inactive and intermediate crystal structures of ShufPTP (Figure 4), the rotamer in the catalytically active conformation of this side chain was chosen to represent reduced cystine. In the case of the unliganded simulations, the catalytic cystine was deprotonated, and in the simulations of the phosphoenzyme intermediate, PO_4_ group was added manually. Both structural modification were handled *via* the CHARMM-GUI.^119^ Further, the oxidized cysteine in the ShufPTP crystal structure pushes out the D63 side chain such that it forms a catalytically non-productive dyad with the E41 side chain (Figure 4). To mitigate this issue, we again used the PyMOL^115^ Mutagenesis Wizard to select the D63 rotamer that was in the closest analog to the position of this side chain in TkPTP as the starting point for our simulations.

All molecular dynamics (MD) simulations were performed using the GPU-accelerated GROMACS 2024.^120^ All simulations were conducted using CHARMM36m^121^ force field and TIP3P water model, at both 300 and 360K (as the enzymes of interest are thermophiles). In total, 18 different systems were simulated: 3 enzyme variants, in both unliganded and phosphoenzyme intermediate states, with the unliganded systems further simulated in P-loop active (low) and inactive (low) conformations (the phosphoenzyme intermediate was only simulated in the P-loop active “low” conformation). For each system, 5 independent trajectories of 500 ns each were propagated using different initial velocities assigned using different random seeds, using a 2 fs time step, leading to a total of 45 μs of cumulative simulation time.

All systems were prepared for simulation using the CHARMM-GUI,^119^ and followed a standard energy minimization, heating (NVT ensemble) and equilibration (NPT ensemble). Production simulations were all performed in the NPT ensemble (1 atm pressure). All simulation analysis was performed using MDAnalysis 2.7.0,^122, 123^ with the exception of non-covalent interaction analysis, which was performed using Key Interaction Networks.^97^

Full details of simulation and analysis protocols are provided in the Supporting Information, and a data package containing sample input files, starting structures, any non-standard parameter files, representative simulation snapshots, and any custom simulation analysis scripts, is provided on Zenodo at the following DOI: 10.5281/zenodo.15074903.

### Empirical Valence Bond Calculations

The rate-limiting hydrolysis of the phosphoenzyme intermediates of ShufPTP and its variant D63N were described using the empirical valence bond (EVB) approach,^96^ following our prior work.^16, 26, 124^ Here, we have performed EVB simulations of the hydrolysis step of the reactions facilitated by either D63 or E132 (D63-as-base and E132-as-base), to compare the energetic barriers of the dominant and proposed backup mechanisms. These reactions were modelled using the valence bond states presented in Figure S19 of ref. ^16^. Note that as both reactions involve proton abstraction by a carboxylate side chain, identical EVB parameters and valence bond states were used to describe the two mechanistic possibilities. Further, we used the same EVB parameters as presented in prior work^125^ for the current calculations.

All EVB simulations were performed using the catalytically active (low) conformation of the ShufPTP P-loop (PDB ID: 5Z5A, 9E9N). The system preparation and initial equilibration for EVB simulations were performed as described in the Supporting Information. Each EVB trajectory was initialized from 20 independent snapshots extracted from the corresponding MD simulations of wild-type and D63N ShufPTP. Starting structures for each EVB trajectory, which are provided on Zenodo (DOI: 10.5281/zenodo.15074903), were selected based on a catalytic distance cutoff of 5Å between the terminal O atoms of D63/E132 and the P atom of the phosphorylated C93 side chain. Each trajectory was first equilibrated for 20 ns at the approximate EVB transition state (*λ* = 0.5), with the subsequent EVB trajectories propagated from the transition state in both the reactant and product directions, as described in our previous work.^16, 125^ Each EVB simulation was performed in 51 individual mapping windows of 200 ps in length per trajectory. This led to a total of 20 ns of equilibration and 10.2 ns EVB simulation time per trajectory, 400 ns equilibration and 204 ns EVB simulation time cumulative per system, and 2μs equilibration time and 1.02 μs EVB simulations over all 5 systems studied (four different starting states / 2 mechanisms for wild-type ShufPTP, and one starting state/mechanism for D63N ShufPTP, as summarized in Table S2).

All EVB simulations were performed using the *Q6* simulation package^126^ and the OPLS-AA^127^ force field, for consistency with previous related studies. All EVB parameters necessary to reproduce our work, as well as a detailed description of the computational methodology and subsequent simulation analysis can be found in the Supporting Information, and on Zenodo, DOI: 10.5281/zenodo.15074903.

## Supplementary Material

Supplement 1

## Figures and Tables

**Table 1.** ShufPTP exhibits comparable turnover numbers to modern PTPs.^a^

Enzyme	*k*_cat_, s^−1^	Reference
YopH	720	^61^
PTP1B	52	^62^
VHZ	3.9	^63^
VHR	3.1	^63^
SsoPTP	3.2	^32^
TkPTP	4.7	^33^
TkPTP D63N	0.01	^33^
ShufPTP	8.6	This work
ShufPTP D63N	0.12	This work

a Data in Table 1 are *k*_cat_ values obtained at the respective pH optimum for each enzyme, at 25 °C except for YopH (22 °C) and TkPTP (20 °C). Data were obtained using the substrate *p*NPP except for TkPTP, where 6,8-difluoro-4-methylumbelliferyl phosphate (DiFMUP) was used. Note that *k*_cat_ reflects the second step of phosphoenzyme hydrolysis, which is substrate independent.

**Table 2.** ShufPTP exhibits a higher rate of inactivation by hydrogen peroxide than other PTPs.

Enzyme	*k*_inact_, M^−1^s^−1^
PTP1^60^	9.1 ± 0.1
VHR^60^	17.9 ± 1.3
LAR^60^	14.0 ± 3.1
YopH^a^	33.0 ± 4.1
PTP1B^a^	18.5 ± 1.6
ShufPTP^a^	64.8 ± 5.0

a Measurements performed in this work.

## References

[1] Wolfenden R.; Snider M. J. The Depth of Chemical Time and the Power of Enzymes as Catalysts. Acc. Chem. Res. 2001, 34, 938–945. 11747411 10.1021/ar000058i

[2] Porter J. L.; Rusli R. A.; Ollis D. L. Directed Evolution of Enzymes for Industrial Biocatalysis. ChemBioChem 2016, 17, 197–203. 26661585 10.1002/cbic.201500280

[3] Badenhorst C. P. S.; Bornscheuer U. T. Getting Momentum: From Biocatalysis to Advanced Synthetic Biology. Trends Biochem. Sci. 2018, 43, 180–198. 29426712 10.1016/j.tibs.2018.01.003

[4] Bornscheuer U. T. The Fourth Wave of Biocatalysis is Approaching. Philos. Trans. A Math. Phys. Eng. Sci.. 2018, 376, 20170063. 29175831 10.1098/rsta.2017.0063

[5] Sandoval B. A.; Hyster T. K. Emerging Strategies for Expanding the Toolbox of Enzymes in Biocatalysis. Curr. Opin. Chem. Biol. 2020, 55, 45–51. 31935627 10.1016/j.cbpa.2019.12.006 PMC7769163

[6] James L. C.; Tawfik D. S. Conformational Diversity and Protein Evolution - A 60 Year Old Hypothesis Revisited. Trends Biochem. Sci. 2003, 28, P361–P368. 10.1016/S0968-0004(03)00135-X 12878003

[7] Tokuriki N.; Tawfik D. S. Protein Dynamism and Evolvability. Science 2009, 324, 203–207. 19359577 10.1126/science.1169375

[8] Campitelli P.; Modi T.; Kumar S.; Banu Ozkan S. The Role of Conformational Dynamics and Allostery in Modulating Protein Evolution. Annu. Rev. Biophys. 2020, 49, 267–288. 32075411 10.1146/annurev-biophys-052118-115517

[9] Crean R. M.; Gardner J. M.; Kamerlin S. C. L. Harnessing Conformational Plasticity to Generate Designer Enzymes. J. Am. Chem. Soc. 2020, 142, 11324–11342. 32496764 10.1021/jacs.0c04924 PMC7467679

[10] Damry A. M.; Jackson C. J. The Evolution and Engineering of Enzyme Activity Through Tuning Conformational Landscapes. Prot. Eng. Des. Sel. 2021, 34, gzab009. 10.1093/protein/gzab009 33903911

[11] Nestl B. M.; Hauer B. Engineering of Flexible Loops in Enzymes. ACS Catal. 2014, 4, 3201–3211.

[12] Corbella M.; Pinto G. P.; Kamerlin S. C. L. Loop Dynamics and the Evolution of Enzyme Activity. Nat. Rev. Chem. 2023, 7, 536–547. 37225920 10.1038/s41570-023-00495-w

[13] Alonso A.; Sasin J.; Bottini N.; Friedberg I.; Friedberg I.; Osterman A.; Godzik A.; Hunter T.; Dixon J.; Mustelin T. Protein Tyrosine Phosphatases in the Human Genome. Cell 2004, 117, 699–711. 15186772 10.1016/j.cell.2004.05.018

[14] Zhang Z.-Y. Chemical and Mechanistic Approaches to the Study of Protein Tyrosine Phosphatases. Acc. Chem. Res. 2003, 36, 385–392. 12809524 10.1021/ar020122r

[15] Moise G.; Morales Y.; Beaumont V.; Caradonna T.; Loria J. P.; Johnson S. J.; Hengge A. C. A YopH PTP1B Chimera Shows the Importance of the WPD-Loop Sequence to the Activity, Structure and Dynamics of Protein Tyrosine Phosphatases. Biochemistry 2018, 57, 5315–5326. 30110154 10.1021/acs.biochem.8b00663

[16] Crean R. M.; Biler M.; van der Kamp M. W.; Hengge A. C.; Kamerlin S. C. L. Loop Dynamics and Enzyme Catalysis in Protein Tyrosine Phosphatases. J. Am. Chem. Soc. 2021, 143, 3830–3845. 33661624 10.1021/jacs.0c11806 PMC8031367

[17] Richard J. P.; Zhai X.; Malabanan M. M. Reflections on the Catalytic Power of a TIM-Barrel. Bioorg. Chem. 2014, 57, 206–212. 25092608 10.1016/j.bioorg.2014.07.001 PMC4256097

[18] Brandão T. A. S.; Johnson S. J.; Hengge A. C. The Molecular Details of WPD-Loop Movement Differ in the Protein-Tyrosine Phosphatases YopH and PTP1B. Arch. Biochem. Biophys. 2012, 525, 53–59. 22698963 10.1016/j.abb.2012.06.002 PMC3422214

[19] Shen R.; Brownless A.-L.; Alansson N.; Corbella M.; Kamerlin S. C. L.; Hengge A. C. SHP-1 Variants Broaden the Understanding of pH-Dependent Activities in Protein Tyrosine Phosphatases. JACS Au 2024, 4, 2874–2885. 39211599 10.1021/jacsau.4c00078 PMC11350601

[20] Cui D. S.; Lipchock J. M.; Brookner D.; Loria J. P. Uncovering the Molecular Interactions in the Catalytic Loop That Modulate the Conformational Dynamics in Protein Tyrosine Phosphatase 1B. J. Am Chem. Soc. 2019, 141, 12634–12647. 31339043 10.1021/jacs.9b04470 PMC8259405

[21] Keng Y. F.; Wu L.; Zhang Z. Y. Probing the Function of the Conserved Tryptophan in the Flexible Loop of the Yersinia Protein-Tyrosine Phosphatase. Eur. J. Biochem. 1999, 259, 809–814. 10092868 10.1046/j.1432-1327.1999.00090.x

[22] Whittier S. K.; Hengge A. C.; Loria J. P. Conformational Motions Regulate Phosphoryl Transfer in Related Protein Tyrosine Phosphatases. Science 2013, 341, 899–903. 23970698 10.1126/science.1241735 PMC4078984

[23] Choy M. S.; Li Y.; Machado L. E. S. F.; Kunze M. B. A.; Connors C. R.; Wei X.; Lindorff-Larsen K.; Page R.; Peti W. Conformational Rigidity and Protein Dynamics at Distinct Timescales Regulate PTP1B Activity and Allostery. Mol. Cell 2017, 65, 644–658.e645. 28212750 10.1016/j.molcel.2017.01.014 PMC5325675

[24] Torgeson K. R.; Clarkson M. W.; Kumar G. S.; Page R.; Peti W. Cooperative Dynamics Across Distinct Structural Elements Regulate PTP1B Activity. J. Biol. Chem. 2020, 295, 13829–13837. 32737198 10.1074/jbc.RA120.014652 PMC7535920

[25] Shen R.; Crean R. M.; Johnson S. J.; Kamerlin S. C. L.; Hengge A. C. Single Residue on the WPD-Loop Affects the pH Dependency of Catalysis in Protein Tyrosine Phosphatases. JACS Au 2021, 5, 646–659. 10.1021/jacsau.1c00054 PMC8297725 34308419

[26] Shen R.; Crean R. M.; Olsen K. J.; Corbella M.; Calixto A. R.; Richan T.; Brandão T. A. S.; Berry R. D.; Tolman A.; Loria J. P.; Insights into the Importance of WPD-Loop Sequence in Protein Tyrosine Phosphatases. Chem. Sci. 2022, 13, 13524–13540. 36507179 10.1039/d2sc04135a PMC9682893

[27] Yang J.; Niu T.; Zhang A.; Mishra A. K.; Zhao Z. J.; Zhou G. W. Relationship Between the Flexibility of the WPD Loop and the Activity of the Catalytic Domain of Protein Tyrosine Phosphatase SHP-1. J. Cell. Biochem. 2001, 84, 47–55. 11746515 10.1002/jcb.1265

[28] Saraste M.; Sibbald P. R.; Wittinghofer A. The P-Loop - A Common Motif in ATP- and GTP-Binding Proteins. Trends Biochem. Sci. 1990, 15, 430–434. 2126155 10.1016/0968-0004(90)90281-f

[29] Smith C. A.; Rayment I. Active Site Comparisons Highlight Structural Similarities Between Myosin and Other P-Loop Proteins. Biophys. J. 1996, 70, 1590–1602. 8785318 10.1016/S0006-3495(96)79745-X PMC1225128

[30] Tabernero L.; Aricescu A. R.; Jones E. Y.; Szdlacsek S. E. Protein Tyrosine Phosphatases: Structure-Function Relationships. FEBS J. 2008, 275, 867–882. 18298793 10.1111/j.1742-4658.2008.06251.x

[31] Pálfy G.; Menyhárd D. K.; Perczel A. Dynamically Encoded Reactivity of Ras Enzymes: Opening New Frontiers for Drug Discovery. Cancer Metastasis Rev. 2020, 39, 1075–1089. 32815102 10.1007/s10555-020-09917-3 PMC7680338

[32] Pinkston J.; Jo J.; Olsen K. J.; Comer D.; Glaittli C. A.; Loria J. P.; Johnson S. J.; Hengge A. C. Significant Loop Motions in the SsoPTP Protein Tyrosine Phosphatase Allow for Dual General Acid Functionality. Biochemistry 2021, 60, 2888–2901. 34496202 10.1021/acs.biochem.1c00365 PMC8561395

[33] Yun H.-Y.; Lee J.; Kim H.; Ryu H.; Shin H.-C.; Oh B.-H.; Ku B.; Kim S. J. Structural Study Reveals the Temperature Dependent Conformational Flexibility of Tk-PTP, a Protein Tyrosine Phosphatase from *Thermococcus Kodakaraensis* KOD1. PLoS One 2018, 13, e0197635. 29791483 10.1371/journal.pone.0197635 PMC5965843

[34] Kennelly P. J. Protein Ser/Thr/Tyr Phosphorylation in the Archaea. J. Biol. Chem. 2014, 289, 9480–9487. 24554702 10.1074/jbc.R113.529412 PMC3974998

[35] Leonard C. J.; Aravind L.; Koonin E. V. Novel Families of Putative Protein Kinases in Bacteria and Archaea: Evolution of the “Eukaryotic” Protein Kinase Superfamily. Genome Res. 1998, 8, 1038–1047. 9799791 10.1101/gr.8.10.1038

[36] Stemmer W. P. C. DNA Shuffling by Random Fragmentation and Reassembly: *In Vitro* Recombination for Molecular Evolution. Proc. Natl. Acad. Sci. USA 1994, 91, 10747–10751. 7938023 10.1073/pnas.91.22.10747 PMC45099

[37] Crameri A.; Raillard S.-A.; Bermudez E.; Stemmer W. P. C. DNA Shuffling of a Family of Genes from Diverse Species Accelerates Directed Evolution. Nature 1998, 391, 288–291. 9440693 10.1038/34663

[38] Ostermeier M.; Shim J. H.; Benkovic S. J. A Combinatorial Approach to Hybrid Enzymes Independent of DNA Homology. Nat. Biotechnol. 1999, 17, 1205–1209. 10585719 10.1038/70754

[39] Price M. T.; Fullerton H.; Moyer C. L. Biogeography and Evolution of Thermococcus isolates from hydrothermal vent systems of the Pacific. Front. Microbiol. 2015, 6, 968. 26441901 10.3389/fmicb.2015.00968 PMC4585236

[40] Salmeen A.; Andersen J. N.; Myers M. P.; Meng T.-C.; Hinks J. A.; Tonks N. K.; Barford D. Redox Regulation of Protein Tyrosine Phosphatase 1B Involves a Sulphenyl-Amide Intermediate. Nature 2003, 423, 769–773. 12802338 10.1038/nature01680

[41] van Montfort R. L. M.; Congreve M.; Tisi D.; Carr R.; Jhoti H. Oxidation State of the Active-Site Cysteine in Protein Tyrosine Phosphatase 1B. Nature 2003, 423, 773–777. 12802339 10.1038/nature01681

[42] Groen A.; Lemeer S.; van der Wijk T.; Overvoorde J.; Heck A. J. R.; Ostman A.; Barford D.; Slijper M.; den Hertog J. Differential Oxiation of Protein-tyrosine Phosphatases. J. Biol. Chem. 2005, 280, 10298–10304. 15623519 10.1074/jbc.M412424200

[43] Weibrecht I.; Böhmer S.-A.; Dagnell M.; Kappert K.; Östman A.; Böhmer F.-D. Oxidation Sensitivity of the Catalytic Cysteine of the Protein-Tyrosine Phosphatases SHP-1 and SHP-2. Free Radic. Biol. Med. 2007, 43, 100–110. 17561098 10.1016/j.freeradbiomed.2007.03.021

[44] Ostman A.; Frijhoff J.; Sandin A.; Böhmer F.-D. Regulation of Protein Tyrosine Phosphatases By Reversible Oxidation. J. Biochem. 2011, 150, 345–356. 21856739 10.1093/jb/mvr104

[45] Wu W.; Hale A. J.; Lemeer S.; den Hertog J. Differential Oxidation of Protein-Tyrosine Phosphatases During Zebrafish Caudal Fin Regeneration. Sci. Rep. 2017, 7, 8460. 28814789 10.1038/s41598-017-07109-8 PMC5559610

[46] Netto L. E. S.; Machado L. E. S. F. Preferential Redox Regulation of Cysteine-Based Protein Tyrosine Phosphatases: Structural and Biochemical Diversity. FEBS J. 2022, 289, 5480–5504. 35490402 10.1111/febs.16466

[47] Tonks N. K. Protein Tyrosine Phosphatases: Mighty Oaks From Little Acorns Grow. IUBMB Life 2023, 75, 337–352. 36971473 10.1002/iub.2716 PMC10254075

[48] Altschul S. F.; Gish W.; Miller W.; Myers E. W.; Lipman D. J. Basic Local Alignment Search Tool. J. Mol. Biol. 1990, 215, 403–410. 2231712 10.1016/S0022-2836(05)80360-2

[49] Sayers E. W.; Bolton E. E.; Brister J. R.; Canese K.; Chan J.; Comeau D. C.; Connor R.; Funk K.; Kelly C.; Kim S.; Database Resources of the National Center for Biotechnology Information. Nucleic Acids Res. 2022, 50, D20–D26. 34850941 10.1093/nar/gkab1112 PMC8728269

[50] Notredame C.; Higgins D. G.; Heringa J. T-Coffee: A Novel Method for Fast and Accurate Multiple Sequence Alignment. J. Mol. Biol. 2000, 302, 205–217. 10964570 10.1006/jmbi.2000.4042

[51] Sharma Y.; Bashir S.; Bhardwaj P.; Ahmad A.; Khan F. Protein Tyrosine Phosphatase SHP-1: Resurgence As New Drug Target for Human Autoimmune Disorders. Immunol. Res. 2016, 64, 804–819. 27216862 10.1007/s12026-016-8805-y

[52] Chu H. M.; Wang A. H. J. Enzyme-Substrate Interactions Revealed by the Crystal Structures of the Archaeal Sulfolobus PTP-fold Phosphatase and its Phosphopeptide Complexes. Proteins 2006, 66, 996–1003. 10.1002/prot.21262 17173287

[53] Haney P. L.; Stees M.; Konisky J. J. Analysis of Thermal Stabilizing Interactions in Mesophilic and Thermophilic Adenylate Kinases from the Genus *Methanococcus*. Biol. Chem. 1999, 274, 28453–28458. 10.1074/jbc.274.40.28453 10497207

[54] Haney P. L.; Badger J. H.; Buldak G. L.; Reich C. L.; Wose C. R.; Olsen G. J. Thermal Adaptation Analyzed by Comparison of Protein Sequences from Mesophilic and Extremely Thermophilic *Methanococcus* Species. Proc. Natl. Acad. Sci. USA 1999, 96, 3578–3583. 10097079 10.1073/pnas.96.7.3578 PMC22336

[55] Melchionna S.; Sinibaldi R.; Briganti G. Explanation of the Stability of Thermophilic Proteins Based on Unique Micromorphology. Biophys. J. 2006, 90, 4204–4212. 16533850 10.1529/biophysj.105.078972 PMC1459513

[56] Gromiha M. M.; Pathak M. C.; Saraboji K.; Ortlund E. A.; Gaucher E. A. Hydrophobic Environment is a Key Factor for the Stability of Thermophilic Proteins. Proteins 2013, 81, 715–721. 23319168 10.1002/prot.24232

[57] Kyte J.; Doolittle R. F. A Simple Method for Displaying the Hydropathic Character of a Protein. J. Mol. Biol. 1981, 157, 105–132. 10.1016/0022-2836(82)90515-0 7108955

[58] Miroshnichenko M. L.; Gongadze G. M.; Rainey F. A.; Kostyukova A. S.; Lysenko A. M.; Chernyh N. A.; Bonch-Osmolovskaya E. A. *Thermococcus gorgonarius sp. nov.* and *Thermococcus pacificus sp. nov.*: Heterotrophic Extremely Thermophilic Archaea from New Zealand Submarine Hot Vents. Int. J. Syst. Bacteriol. 1998, 48, 23–29. 9542072 10.1099/00207713-48-1-23

[59] Zhang Z. Y.; Malachowski W. P.; van Etten R. L.; Dixon J. E. Nature of the Rate-Determining Steps of the Reaction Catalyzed by the Yersinia Protein-Tyrosine Phosphatase. J. Biol. Chem. 1994, 269, 8140–8145. 8132539

[60] Denu J. M.; Tanner K. G. Specific and Reversible Inactivation of Protein Tyrosine Phosphatases By Hydrogen Peroxide: Evidence For a Sulfenic Acid Intermediate and Implications for Redox Regulation. Biochemistry 1998, 37, 5633–5642. 9548949 10.1021/bi973035t

[61] Moise G.; Gallup N. M.; Alexandrova A. N.; Hengge A. C.; Johnson S. J. Conservative Tryptophan Mutants of the Protein Tyrosine Phosphatase YopH Exhibit Impaired WPD-Loop Function and Crystallize with Divanadate Esters in Their Active Sites. Biochemistry 2015, 54, 6490–6500. 26445170 10.1021/acs.biochem.5b00496 PMC4887194

[62] Brandão T. A. S.; Hengge A. C.; Johnson S. J. Insights into the Reaction of Protein-Tyrosine Phosphatase 1B. J. Biol. Chem. 2010, 285, 15874–15883. 20236928 10.1074/jbc.M109.066951 PMC2871455

[63] Kuznetsov V. I.; Hengge A. C. New Functional Aspects of the Atypical Protein Tyrosine Phosphatase VHZ. Biochemistry 2013, 52, 8012–8025. 24073992 10.1021/bi400776z PMC3856698

[64] Zhang Z. Y.; Wang Y.; Dixon J. E. Dissecting the Catalytic Mechanism of Protein-Tyrosine Phosphatases. Proc. Natl. Acad. Sci. USA 1994, 91, 1624–1627. 8127855 10.1073/pnas.91.5.1624 PMC43215

[65] Denu J. M.; Zhou G.; Guo Y.; Dixon J. E. The Catalytic Role of Aspartic Acid-92 in the Human Dual-Specific Protein-Tyrosine-Phosphatase Vaccinia H1-Related. Biochemistry 1995, 34, 3396–3403. 7880835 10.1021/bi00010a031

[66] Robertson A. D.; Murphy K. P. Protein Structure and the Energetics of Protein Stability. Chem. Rev. 1997, 97, 1251–1268. 11851450 10.1021/cr960383c

[67] Lumry R.; Eyring H. Conformation Changes in Proteins. J. Phys. Chem. 1954, 58, 110–120.

[68] Klibanov A. M.; Ahern T. J. Thermal Stability of Proteins.; Alan R. Liss, 1987.

[69] Sanchez-Ruiz J. M. Theoretical Analysis of Lumry-Eyring Models in Differential Scanning Calorimetry. Biophys. J. 1992, 61, 921–935. 19431826 10.1016/S0006-3495(92)81899-4 PMC1260351

[70] Zhang Z. Y. Kinetic and Mechanistic Characterization of a Mammalian Protein-Tyrosine Phosphatase, PTP1. J. Biol. Chem. 1995, 270, 11199–11204. 7744751 10.1074/jbc.270.19.11199

[71] Chen L.; Montserat J.; Lawrence D. S.; Zhang Z. Y. VHR and PTP1 Protein Phosphatases Exhibit Remarkably Different Active Site Specificities Toward Low Molecular Weight Nonpeptidic Substrates. Biochemistry 1996, 35, 9349–9354. 8755712 10.1021/bi960700+

[72] Howell L. D.; Griffiths C.; Slade L. W.; Potts M.; Kennelly P. J. Substrate Specificity of IphP, a Cyanobacterial Dual-Specificity Protein Phosphatase With MAP Kinase Phosphatase Activity. Biochemistry 1996, 35, 7566–7572. 8652537 10.1021/bi9600409

[73] Savle P. S.; Shelton T. E.; Meadows C. A.; Potts M.; Gandour R. D.; Kennelly P. J. N-(Cyclohexanecarboxyl)-O-Phospho-L-Serine, a Minimal Substrate for the Dual-Specificity Protein Phosphatase IphP. Arch. Biochem. Biophys. 2000, 376, 439–448. 10775432 10.1006/abbi.2000.1750

[74] Mukhopadhyay A.; Kennelly P. J. A Low Molecular Weight Protein Tyrosine Phosphatase From *Synechocystis sp.* Strain PCC 6803: Enzymatic Characterization and Identification of Its Potential Substrates. J. Biochem. 2011, 149, 551–562. 21288886 10.1093/jb/mvr014 PMC3115683

[75] Pinkston J.; Shen R.; Simons C. R.; Hengge A. C. Competitive Measurement of β/α Naphthyl Phosphate Catalytic Efficiency By Phosphatases Utilizing Quantitative NMR. Anal. Chem. 2022, 651, 114727. 10.1016/j.ab.2022.114727 35580735

[76] Claiborne A.; Miller H.; Parsonage D.; Ross R. P. Protein-Sulfenic Acid Stabilization and Function in Enzyme Catalysis and Gene Regulation. FASEB J. 1993, 7, 1483–1490. 8262333 10.1096/fasebj.7.15.8262333

[77] Salmeen A.; Barford D. Functions and Mechanisms of Redox Regulation and Cysteine-Based Phosphatases. Antioxid. Redox. Signal. 2005, 7, 560–577. 15890001 10.1089/ars.2005.7.560

[78] Burgoyne J. R.; Madhani M.; Cuello F.; Charles R. L.; Brennan J. P.; Schroder E.; Browning D. D.; Eaton P. Cysteine Redox Sensor in PKGla Enables Oxidant-Induced Activation. Science 2007, 317, 1393–1397. 17717153 10.1126/science.1144318

[79] Brandes N.; Schmitt S.; Jakob U. Thiol-Based Redox Switches in Eukaryotic Proteins. Antioxid. Redox. 2009, 11, 997–1014. 10.1089/ars.2008.2285 PMC2787739 18999917

[80] Klomsiri C.; Karplus P. A.; Poole L. B. Cysteine-Based Redox Switches in Enzymes. Antioxid. Redox. Signal. 2011, 14, 1065–1077. 20799881 10.1089/ars.2010.3376 PMC3064533

[81] Gonzalez-Rubio M.; Voit S.; Rodriguez-Puyol D.; Weber M.; Marx M. Oxidative Stress Induces Tyrosine Phosphorylation of PDGF Alpha- and Beta-Receptors and pp60c-Src in Mesangial Cells. Kidney Int. 1996, 50, 164–173. 8807585 10.1038/ki.1996.299

[82] Flohe L.; Brigelius-Flohe R.; Saliou C.; Traber M. G.; Packer L. Redox Regulation of NF-Kappa B Activation. Free Radic. Biol. Med. 1997, 22, 1115–1126. 9034250 10.1016/s0891-5849(96)00501-1

[83] Suzuki Y. J.; Forman H. J.; Sevanian A. Oxidants as Stimulators of Signal Transduction. Free Radic. Biol. Med. 1997, 22, 269–285. 8958153 10.1016/s0891-5849(96)00275-4

[84] Krejsa C. M.; Nadler S. G.; Esselstyn J. M.; Kavanagh T. J.; Ledbetter J. A.; Schieven G. L. Role of Oxidative Stress in the Action of Vanadium Phosphotyrosine Phosphatase Inhibitors. Redox Independent Activation of NF-KappaB. J. Biol. Chem. 1997, 272, 11541–11549. 9111069 10.1074/jbc.272.17.11541

[85] Denu J. M.; Dixon J. E. Protein Tyrosine Phosphatases: Mechanisms of Catalysis and Regulation. Curr. Opin. Chem. Biol. 1998, 2, 633–641. 9818190 10.1016/s1367-5931(98)80095-1

[86] Caselli A.; Marzocchini R.; Camici G.; Manao G.; Moneti G.; Pieraccini G.; Ramponi G. The Inactivation Mechanism of Low Molecular Weight Phosphotyrosine-Protein Phosphatase by H_2_O_2_. J. Biol. Chem. 1998, 273, 32556–32560. 10.1074/jbc.273.49.32554 9829991

[87] Sohn J.; Rudolph J. Catalytic and Chemical Competence of Regulation of Cdc25 Phosphatase By Oxidation/Reduction. Biochemistry 2003, 42, 10060–10070. 12939134 10.1021/bi0345081

[88] Tonks N. K. Redox Redux: Revisiting PTPs and the Control of Cell Signaling. Cell 2005, 121, 667–670. 15935753 10.1016/j.cell.2005.05.016

[89] Yang J.; Groen A.; Lemeer S.; Jans A.; Slijper M.; Roe S. M.; den Hertog J.; Barford D. Reversible Oxidation of the Membrane Distal Domain of Receptor PTPalpha Is Mediated By Aa Cyclic Sulfenamide. Biochemistry 2007, 46, 709–719. 17223692 10.1021/bi061546m

[90] Lou Y. W.; Chen Y. Y.; Hsu S. F.; Chen R. K.; Lee C. L.; Khoo K. H.; Tonks N. K.; Meng T. C. Redox Regulation of the Protein Tyrosine Phosphatase PTP1B In Cancer Cells. FEBS J. 2008, 275, 69–88. 18067579 10.1111/j.1742-4658.2007.06173.x

[91] Chen C. Y.; Willard D.; Rudolph J. Redox Regulation of SH2-Domain-Containing Protein Tyrosine Phosphatases By Two Backdoor Cysteines. Biochemistry 2009, 48, 1399–1409. 19166311 10.1021/bi801973z

[92] Tautz L.; Critton D. A.; Grotegut S. Protein Tyrosine Phosphatases: Structure, Function, and Implication in Human Disease. Methods Mol. Biol. 2013, 1053, 179–221. 23860656 10.1007/978-1-62703-562-0_13 PMC8158066

[93] Ben-David M.; Elias M.; Filippi J.-J.; Duñach E.; Silman I.; Sussman J. L.; Tawfik D. S. Catalytic Versatility and Backups in Enzyme Active Sites: The Case of Serum Paraoxonase 1. J. Mol. Biol. 2012, 418, 181–196. 22387469 10.1016/j.jmb.2012.02.042

[94] Ben-David M.; Soskine M.; Dubovetskyi A.; Cherukuri K.-P.; Dym O.; Sussman J. L.; Liao Q.; Szeler K.; Kamerlin S. C. L.; Tawfik D. S. Enzyme Evolution: An Epistatic Ratchet versus a Smooth Reversible Transition. Mol. Biol. Evol. 2020, 37, 1133–1147. 31873734 10.1093/molbev/msz298

[95] Corbella M.; Bravo J.; Demkiv A.; Calixto A. R.; Elias M.; Kamerlin S. C. L. Catalytic Redundancies and Conformational Plasticity Drives Selectivity and Promiscuity in Quorum Quenching Lactonases. JACS Au 2024, 4, 3519–3536. 39328773 10.1021/jacsau.4c00404 PMC11423328

[96] Warshel A.; Weiss R. M. An Empirical Valence Bond Approach for Comparing Reactions in Solutions and in Enzymes. J. Am. Chem. Soc. 1980, 102, 6218–6226.

[97] Yehorova D.; Crean R. M.; Kasson P. M.; Kamerlin S. C. L. Key Interaction Networks: Identifying Evolutionarily Conserved Non-Covalent Interaction Networks Across Protein Families. Prot. Sci. 2024, 33, e4911. 10.1002/pro.4911 PMC10868456 38358258

[98] Demkiv A. O.; Toledo-Patiño S.; Medina-Carmona E.; Berg A.; Pinto G. P.; Parracino A.; Sanchez-Ruiz J. M.; Hengge A. C.; Laurino P.; Longo L. M.; Redefining the Limits of Functional Continuity in the Early Evolution of P-Loop NTPases. Mol. Biol. Evol. 2025, Advance Article. 10.1093/molbev/msaf055 PMC11959459 40070202

[99] Torgeson K. R.; Clarkson M. W.; Granata D.; Lindorff-Larsen K.; Page R.; Peti W. Conserved Conformational Dynamics Determine Enzyme Activity. Sci. Adv. 2022, 8, eabo5546. 35921420 10.1126/sciadv.abo5546 PMC9348788

[100] Tonks N. K. PTP1B: From the Sidelines to the Front Lines! FEBS Lett. 2003, 546, 140–148. 12829250 10.1016/s0014-5793(03)00603-3

[101] Barrios A. M. PTPs: Degrading the Undruggable. J. Med. Chem. 2020, 63, 7508–7509. 32615037 10.1021/acs.jmedchem.0c01000

[102] Jeon S.-J.; Fujiwara S.; Takagi M.; Tanaka T.; Imanaka T. Tk-PTP, Protein Tyrosine/Serine Phosphatase from Hyperthermophilic Archaeon *Thermococcus kodakaraensis* KOD1: Enzymatic Characteristics and Identification of its Substrate Properties. Biochem. Biophys. Res. Commun. 2002, 295, 508–514. 12150979 10.1016/s0006-291x(02)00705-2

[103] Rampelotto P. H. Extremophiles and Extreme Environments. Life 2013, 3, 482–485. 25369817 10.3390/life3030482 PMC4187170

[104] Littlechild J. A. Archaeal Enzymes and Applications in Industrial Biocatalysis. Archaea 2015, 2015, 147671. 26494981 10.1155/2015/147671 PMC4606452

[105] Cabrera M. A.; Blamey J. M. Biotechnological Applications of Archaeal Enzymes from Extreme Environments. Biol. Res. 2018, 51, 37. 30290805 10.1186/s40659-018-0186-3 PMC6172850

[106] Bräsen C.; Esser D.; Rauch B.; Siebers B. Carbohydrate Metabolism in *Archaea*: Current Insights into Unusual Enzymes and Pathways and Their Regulation. Microbiol. Mol. Biol. Rev. 2014, 78, 89–175. 24600042 10.1128/MMBR.00041-13 PMC3957730

[107] Schiraldi C.; Giuliano M.; De Rosa M. Perspectives in Biotechnological Applications of Archaea. Archaea 2002, 1, 75–86. 15803645 10.1155/2002/436561 PMC2685559

[108] Straub C. T.; Counts J. A.; Nguyen D. M. N.; Wu C.-H.; Zeldes B. M.; Crosby J. R.; Conway J. M.; Otten J. K.; Lipscomb G. L.; Schut G. J.; Biotechnology of Extremely Thermophilic Archaea. FEMS Microbiol. Rev. 2018, 42, 543–578. 29945179 10.1093/femsre/fuy012 PMC6454523

[109] Pfeifer K.; Ergal I.; Koller M.; Basen M.; Schuster B.; Rittmann S. K.-M. R. Archaea Biotechnology. Biotechnol. Adv. 2021, 47, 107668. 33271237 10.1016/j.biotechadv.2020.107668

[110] Aparici-Carratalá D.; Esclapez J.; Bautista V.; Bonete M.-J.; Camacho M. Archaea: Current and Potential Biotechnological Applications. Res. Microbiol. 2023, 174, 104080. 37196775 10.1016/j.resmic.2023.104080

[111] Hengge A. C.; Sowa G. A.; Wu L.; Zhang Z.-Y. Nature of the Transition State of the Protein-Tyrosine Phosphatase-Catalyzed Reaction. Biochemistry 1995, 34, 13892–13987. 10.1021/bi00043a003 7577995

[112] Minor W.; Cymorowski M.; Otwinowski Z.; Chruszcz M. *HKL*-3000: The Integration of Data Reduction and Structure Solution - From Diffraction Images to an Initial Model in Munites. Acta. Cryst. D Struct. Biol. 2006, 62, 859–866. 10.1107/S0907444906019949 16855301

[113] Afonine P. V.; Grosse-Kunstleve R. W.; Echols N.; Headd J. J.; Moriarty N. W.; Mustyakimov M.; Terwilliger T. C.; Urzhumtsev A.; Zwart P. H.; Adams P. D. Towards Automated Crystallographic Structure Refinement With phenix.refine. Acta Crystallogr. D Biol. Crystallogr. 2012, 68, 352–367. 22505256 10.1107/S0907444912001308 PMC3322595

[114] Emsley P.; Lohkamp B.; Scott W. G.; Cowtan K. Features and Development of Coot. Acta Crystallogr. D Biol. Crystallogr. 2010, 66, 486–501. 20383002 10.1107/S0907444910007493 PMC2852313

[115] The PyMOL Molecular Graphics System. Version 3.0. Schrödinger, LLC.

[116] Rodriguez-Larrea D.; Minning S.; Borchert T. V.; Sanchez-Ruiz J. M. Role of Solvation Barriers in Protein Kinetic Stability. J. Mol. Biol. 2006, 360, 715–724. 16784752 10.1016/j.jmb.2006.05.009

[117] Cleland W. W. Determining the Chemical Mechanisms of Enzyme-Catalyzed Reactions by Kinetic Studies. Enzymol. Relat. Areas Mol. Biol. 1977, 45, 273–387. 10.1002/9780470122907.ch4 21524

[118] Pinkston J.; Shen R.; Simons C. R.; Hengge A. C. Competitive Measurement of β/α Naphthyl Phosphate Catalytic Efficiency by Phosphatases Utilizing Quantitative NMR. Anal. Biochem. 2022, 651, 114727. 35580735 10.1016/j.ab.2022.114727

[119] Jo S.; Kim T.; Iyer V. G.; Im W. CHARMM-GUI: A Web-based Graphical User Interface for CHARMM. J. Comput. Chem. 2008, 29, 1859–1865. 18351591 10.1002/jcc.20945

[120] van der Spoel D.; Lindahl E.; Hess B.; Groenhof G.; Mark A. E.; Berendsen H. J. C. GROMACS: Fast, Flexible and Free. J. Comp. Chem. 2005, 26, 1701–1718. 16211538 10.1002/jcc.20291

[121] Huang J.; Rauscher S.; Nawrocki G.; Ran T.; Feig M.; de Groot B. L.; Grubmüller H.; MacKerell A. D. Jr. CHARMM36m: An Improved Force Field for Folded and Intrinsically Disordered Proteins. Nat. Methods. 2016, 14, 71–73. 27819658 10.1038/nmeth.4067 PMC5199616

[122] Gowers R.; Linke M.; Barnoud J.; Reddy T.; Melo M.; Seyler S.; Domanski J.; Dotson D.; Buchoux S.; Kenney I.; MDAnalysis: A Python Package for the Rapid Analysis of Molecular Dynamics Simulations. Proc. 15th Python Sci. Conf. 2016.

[123] Michaud-Agrawal N.; Denning E. J.; Woolf T. B.; Beckstein O. MDAnalysis: A Toolkit for the Analysis of Molecular Dynamics Simulations. J. Comp. Chem. 2011, 32, 2319–2327. 21500218 10.1002/jcc.21787 PMC3144279

[124] Crean R.; Corbella M.; Calixto A. R.; Hengge A.; Kamerlin S. C. L. Sequence – Dynamics – Function Relationships in Protein Tyrosine Phosphatases. QRB Discov. 2024, 5, e4. 38689874 10.1017/qrd.2024.3 PMC11058592

[125] Crean R. M.; Biler M.; Corbella M.; Calixto A. R.; van der Kamp M.; Hengge A. C.; Kamerlin S. C. L. Correction to “Loop Dynamics and Enzyme Catalysis in Protein Tyrosine Phosphatases”. J. Am. Chem. Soc. 2022, 144, 10091–10093. 35609280 10.1021/jacs.2c04624 PMC11027752

[126] Bauer P.; Barrozo A.; Purg M.; Amrein B. A.; Esguerra M.; Wilson P. B.; Major D. T.; Åqvist J.; Kamerlin S. C. L. Q6: A Comprehensive Toolkit for Empirical Valence Bond and Related Free Energy Calculations. SoftwareX 2018, 7, 388–395.

[127] Jorgensen W. L.; Maxwell D. S.; Tirado-Rives J. Development and Testing of the OPLS All-Atom Force Field on Conformational Energetics and Properties of Organic Liquids. J. Am. Chem. Soc. 1996, 118, 11225–11236.

[128] Boerner T. J.; Deems S.; Furlani T. R.; Knuth S. L.; Towns J. ACCESS: Advancing Innovation: NSF’s Advanced Cyberinfrastructure Coordination Ecosystem: Services & Support. In In Practice and Experience in Advanced Research Computing (PEARC ’23), Portland, OR, USA, 23–27, 2023, 2023; ACM, New York, NY, USA.

