# Supplementary material for: Conformational Dynamics and Catalytic Backups in a Hyper-Thermostable Engineered Archaeal Protein Tyrosine Phosphatase
Source: bioRxiv. 2025 Mar 26:2025.03.26.645524. Preprint. [Version 1] doi: 10.1101/2025.03.26.645524 (PMC11974932; doi:10.1101/2025.03.26.645524)
Supplement: Supplement 1 [file media-1.pdf]

Supporting Information for:

Conformational Dynamics and Catalytic Backups in a Hyper-Thermostable Engineered Archaeal Protein Tyrosine Phosphatase

Dariia Yehorova,[1,†] Nikolas Alansson,[1,†] Ruidan Shen,[2,†] Joshua M. Denson,[2,†] Michael Robinson,[3] Valeria A. Risso,[4] Nuria Ramirez Molina,[5] J. Patrick Loria,[6,7] Eric A. Gaucher,[5] Jose M. Sanchez-Ruiz,[4] Alvan C. Hengge,[2,*] Sean J. Johnson[2,*] and Shina C. L. Kamerlin[1,8,*]

1. School of Chemistry and Biochemistry, Georgia Institute of Technology, 901 Atlantic Drive NW, Atlanta, GA 30332, USA.
2. Department of Chemistry & Biochemistry, Utah State University, 0300 Old Main Hill, Logan, UT 84322-0300, USA.
3. Department of Chemistry – BMC, Uppsala University, BMC Box 576, S-751 23 Uppsala, Sweden.
4. Departamento de Química Física, Facultad de Ciencias, Unidad de Excelencia de Química Aplicada a Biomedicina y Medioambiente (UEQ), Universidad de Granada, Granada, 18071, Spain.
5. Department of Biology, Georgia State University, 100 Piedmont Ave SE, Atlanta, GA 30303, USA.
6. Department of Chemistry, Yale University, P. O. Box 208107, New Haven, CT, 06520-8107.
7. Department of Molecular Biophysics and Biochemistry, Yale University, New Haven, CT, 06520, USA.
8. Department of Chemistry, Lund University, Box 124, 22100 Lund, Sweden.

[†] These authors contributed equally to this work.

Corresponding Author Email Addresses: alvan.hengge@usu.edu, sean.johnson@usu.edu, skamerlin3@gatech.edu

Table of Contents

S1. Computational Methodology S3

Molecular Dynamics Simulations S3

Molecular Dynamics Analysis S4

Empirical Valence Bond Simulations S6

S2. Supplementary Figures S8

S3. Supplementary Tables S19

S4. Supplementary References S21

S1. Computational Methodology

Molecular Dynamics Simulations

Molecular dynamics (MD) simulations of TkPTP, ShufPTP, and the ShufPTP D63N variant, were performed using GPU-accelerated GROMACS 2024,[1] with system preparation performed as described in the main text. Enzymes with the active (low) P-loop form (PDB IDs: 5Z5A[2] and 9E9N) were simulated in the unliganded and phosphoenzyme intermediate states, while crystal structures with the inactive (high) (PDB IDs: 5Z59[2] and 9E9U) and intermediate (PDB IDs: 9E9L, 9E9M) P-loop were simulated in their unliganded state. To account for temperature effects, simulations were performed at both 300 and 360K in the NPT ensemble (1atm), leading to 18 discrete simulation systems.

All simulations were performed using the CHARMM36m force field,[3] and the TIP3P water model,[4] using a common equilibration and production protocol described below. Each system was simulated for 5 x 500 ns of production using different initial velocities (assigned using different random seeds) per trajectory, leading to a total of 45 μs of cumulative simulation time over 18 systems. System preparation was performed using the standard protocol of the CHARMM-GUI[5] builder, with the phosphoenzyme intermediate parameterized using CHARMM-GUI solution builder tools. The parameters for the phosphoenzyme intermediate are presented in the associated Zenodo data package, DOI: 10.5281/zenodo.15074903. Each system was solvated in a cubic box of 67x67x67Å or 10Å from the edge boundary of the protein with 0.15M NaCl added to the system.

All systems were equilibrated using a common protocol, in the following steps: (1) 5000 steps of initial energy minimization using the steepest descent algorithm. (2) Gradual heating of the system from 100 to 300K (or 360K) over the course of 1.25 ns of simulation time in the NVT

ensemble, during which backbone heavy atoms were restrained using 400 kJ mol^{-1} Å^{-2} harmonic restraints, while sidechain atoms were restrained using 40 kJ mol^{-1} Å^{-2} harmonic restraints. (3) A further 1 ns simulation in the NPT ensemble, in which the backbone heavy atom restraints were reduced to 100 kJ mol^{-1} Å^{-2}, and the side chain restraints were removed. (4) The backbone restraints were dropped again to 50 kJ mol^{-1} Å^{-2} for another 1ns long NPT simulation. (5) A 1 ns long MD simulation without restraints was performed to complete the equilibration. Finally, 500 ns of production MD was performed per trajectory, in an NPT (1atm) ensemble. Simulation equilibration is shown in **Figures S10** to **S12**.

A 1 fs timestep was used for all equilibration steps, followed by a 2 fs time step for production MD. All NVT and NPT simulations used velocity rescaling[6] for temperature coupling, and all NPT simulations used a stochastic cell rescaling barostat[7] to control pressure. Both NVT and NPT equilibration simulations used the LINCS algorithm[8] to restrain all bonds to hydrogen atoms. All simulation steps used a 10Å non-bonded interaction cut-off to evaluate long-range electrostatic interactions, using the Verlet non-bonded cutoff scheme,[9] with long range electrostatics being evaluated using the Particle Mesh Ewald (PME) algorithm.[10] Finally, snapshots were saved every 100ps of simulation time for further analysis.

Molecular Dynamics Analysis

Analysis of loop dynamics and catalytic distances observed during our MD simulations was performed using MDAnalysis 2.7.0.[11, 12] Root mean square deviation (RMSD)[13] and fluctuation (RMSF)[14] analysis for all systems was conducted using backbone and C_{α}-atoms, respectively. Further, RMSF values were visualized using simulation snapshots obtained every 5ns of simulation time, where the residue color is altered to represent a corresponding RMSF value *via* B-factor visualization in PyMOL 3.0.3.[15]

P-loop conformational analysis was performed by extracting the (ϕ, ψ) angles of each residue in the P-loop (residues 94-99) from every 100ps of the MD simulations initialized from the P-loop active/low (PDB: 5Z5A[2] for TkPTP, and 9E9N for ShufPTP), intermediate (PDB: 9E9L and 9E9M for ShufPTP) and inactive/high (PDB: 5Z59[2] for TkPTP, and 9E9U for ShufPTP) conformations. From this, an "angle RMSD" was calculated to assess the similarity between simulation frames extracted from the MD simulations and the corresponding crystallographic structure according to Equation 1, as described in the main text.

All distance analysis was performed by computing a minimum distance between the phosphorus atom of the phosphorylated C93 at the intermediate state, and the closest oxygen atom on the carboxylic acid side chain of the assessed residues (E38, E39, E41, D63, E132). Quantification of the presence of a potentially nucleophilic water molecule was performed by identifying the number of frames in which the O-P distance is less than 6Å, and a water molecule is located within 3.5Å of the carboxylic O and phosphate P atoms.

Finally, non-covalent interaction analysis was conducted using Key Interaction Networks.[16] The interactions and their conservation scores in the active unphosphorylated simulations were calculated and compared between TkPTP and ShufPTP to identify uniquely stabilizing interactions.

A data package containing sample input files, starting structures, any non-standard parameter files, representative simulation snapshots, and any custom simulation analysis scripts is provided on Zenodo at the following DOI: 10.5281/zenodo.15074903.

Empirical Valence Bond Simulations

System Preparation for the Empirical Valence Bond Simulations

Empirical valence bond (EVB) simulations[17] were performed to characterize the second, rate-limiting hydrolysis step of the reaction to analyze the relative catalytic roles of D63 located on the Acid-loop and E132 located on the Q-loop of ShufPTP. This analysis was carried out for ShufPTP and its variant D63N, with a closed IPD-loop, and the P-loop in the active (low) conformation (PDB: 9E9N). To account for the dynamic nature of the electronic environment at the active site of the enzyme, we assessed the five following scenarios (**Figure S6**): the reaction in ShufPTP is catalyzed by D63 while E132 is located within or outside of the catalytic distance cutoff, the reaction is catalyzed by E132 while D63 is located within or outside of the catalytic distance cutoff, and the reaction in variant D63N, where E132 is the only remaining reactive residue (due to elimination of D63). All systems were simulated with 20 replicas, initialized from the frames of the MD simulations of phosphorylated ShufPTP with active (low) P-loop. Frames were selected using MDAnalysis, while setting the catalytic distance cutoff to 5Å. The reactive water was manually adjusted in each selected frame to ensure a catalytically productive conformation. Simulation starting structures can be found in the associated Zenodo package, DOI: 10.5281/zenodo.15074903.

All systems were solvated in a 23 Å radius droplet of TIP3P23 water molecules, centered on the P atom of phosphorylated C93 of the P-loop, and described using the Surface Constrained All Atom Solvent (SCAAS) model,[18] as in our previous work.[19-21] In this model, all residues within the inner 85% of the sphere are fully mobile during the simulations, all residues within the outer 15% of the sphere are restrained to their original crystallographic positions using 10 kcal mol^{-1} Å^{-2} harmonic positional restraints, and all residues outside the water droplet are constrained to their

crystallographic coordinates using 200 kcal mol^{-1} $Å^{-2}$ harmonic positional restraints. Residues outside the water droplet and in the restrained region of the droplet were simulated in their uncharged forms, in order to avoid introducing system instabilities associated with the presence of charged residues outside the explicit simulation sphere. A complete list of the assigned protonation states of all ionizable residues in our EVB calculations, as well as the histidine tautomerization states assigned, are provided in **Table S3**. All EVB parameters used in this study follow our prior work,[22] and have been provided in the associated Zenodo data package, DOI: 10.5281/zenodo.15074903.

System Equilibration and Empirical Valence Bond Simulations

All EVB simulations in this work were performed using the *Q6*[23] simulation package, using the OPLS-AA[24] force field as implemented into Q6. A 10 Å cut-off was set for all non-bonded interactions, except those involved in the chemical reactions, for which the cut-off was set to 99 Å (effectively no cut-off). Long-range electrostatic interactions were described using the local reaction field (LRF)[25] approach, and the temperature was controlled by the Berendsen thermostat.[26] All systems underwent a sequence of short equilibration and heating simulations over a total of 213 ps of simulation time, in order to gradually remove possible steric clashes and bad contacts in the system prior to equilibration at 300 K. Once the system reached 300 K, all restraints on the mobile region of the protein were removed, and only weak (0.5 kcal mol^{-1} $Å^{-2}$) harmonic positional restraints were retained on the reacting moieties. Finally, we performed a further 20 ns of equilibration. Finally, weak harmonic positional restraints of 0.5 kcal mol^{-1} $Å^{-2}$ placed on the reactive atoms during the equilibration were retained for the production EVB simulations as well. All further analysis of the data was done using *Q*tools v0.5.10 (Zenodo DOI: 10.5281/zenodo.842003).

S2. Supplementary Figures

```
WP_088884773-1      MWPSARFVDESVAFSRMPTEGELDEVARDFDTIVVLVEDYELPYSLEEWEKRGVEVLHSP
WP_088856889-1      MWPSARFVDDNVAFSRMPTRRELDGIASDFDAVVVLVEEYELPYRLEEWEKRGIEVLHSP
WP_088865855-1      MWRSAKFIDDNVAFSRMPARSEIDEAAETFDAVVVLVEEFELPYSLSEWQKRGVEVLHSP
HDZ36498-1          MWRSAKFIDDNVAFSRMPTASEIAEVAKSFDAVVVLVEEFELPYSLDEWKKRGVEVLHSP
WP_058937812-1      MWRPARFVDGNVAFSRMPTRREMDEVAKTFDAVVVLVEEFELPYGLDEWWKQGVEVLHSP
Shuffled            MWPSARFVDDNVAFSRMPTERELDEVAKDFDAVVVLVEEYELPYSLEEWKKRGVEVLHSP
                    ** .*:*:* .*******:  *:   *  **::*****::**** *.** *:*:******

WP_088884773-1      IPDFTAPSLEQLLEILRWIDARVHEGKRVLIHCMGGLGRSGTVAVAWLIYSKGLPLREAL
WP_088856889-1      IEDFTAPTLNQLLEILEWIERRVGEGKKVLIHCLGGLGRSGTIATAWLMYSKGLRLQEAL
WP_088865855-1      IRDFSAPALDQLLKILRWIEARVAEGKKVLIHCMGGLGRSGTVAVAWVMYSKGLPLREAL
HDZ36498-1          VRDFSAPALNQLLEILRWIEERVREGKKVLIHCMGGLGRSGTVAVAWLMYAKGLPLREAL
WP_058937812-1      IEDFTAPSLSQLLEILHWIEARVEEGKKVLIHCMGGLGRSGTVAVAWVMYSKGLPMREAL
Shuffled            IPDFTAPSLEQLLEILRWIEARVREGKKVLIHCMGGLGRSGTVAVAWLMYSKGLPLREAL
                    : **:**:*.***:**.**: ** ***:*****:********:*.**::*:*** ::***

WP_088884773-1      RRVRSLRPGAVETYEQMEVLGELKGFLRSR
WP_088856889-1      RRVRSIRPGSVETYEQMEVLKELEKLLRSR
WP_088865855-1      RRVRRVRPGAVEVEEQMGVLKELERLLRSR
HDZ36498-1          GRVRMVRPGAVEIWEQMKVLKELERLLRSR
WP_058937812-1      KMVRRIRPGAVEVEEQRGVLKELERLLRSR
Shuffled            RRVRSLRPGAVETYEQMEVLKELEKFLRSR
                      ** :***:**  **  ** **: :****
```

Figure S1. Multiple sequence alignment of protein sequences used for random shuffling generation of ShufPTP. Sequences were obtained from GenBank.[27] Sequences correspond to a dual specificity protein phosphatase from *Thermococcus gorganarius* (WP_088884773-1), *Thermococcus siculi* (WP_088856889-1), *Thermococcus radiotolerans* (WP_088865855-1), *Thermococcus sp.* (uncharacterized, HDZ-36498-1), and *Thermococcus celericrescens* (WP_058937812-1).

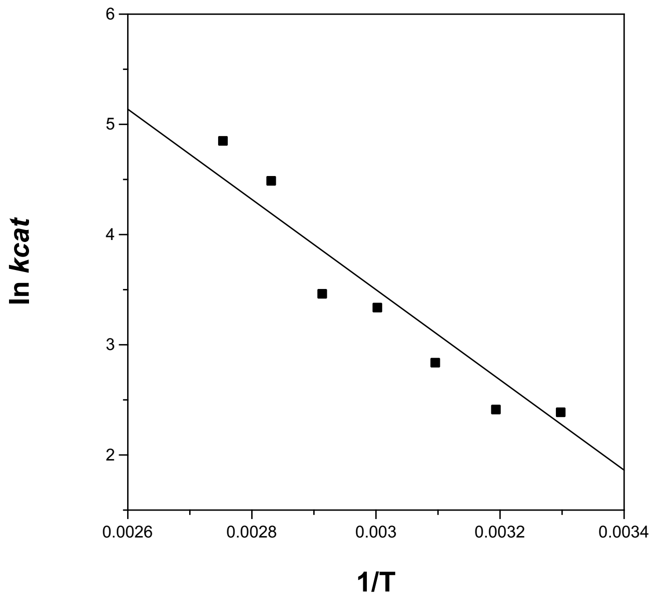

Figure S2. **Thermal stability of ShufPTP.** The Arrhenius plot for ShufPTP-catalyzed hydrolysis of *p*NPP in the range of 22 - 90 °C indicates high thermostability up to 90 °C. Loss of activity at high temperatures would result in downward curvature at the highest temperatures (the left side of the graph), which is not observed.

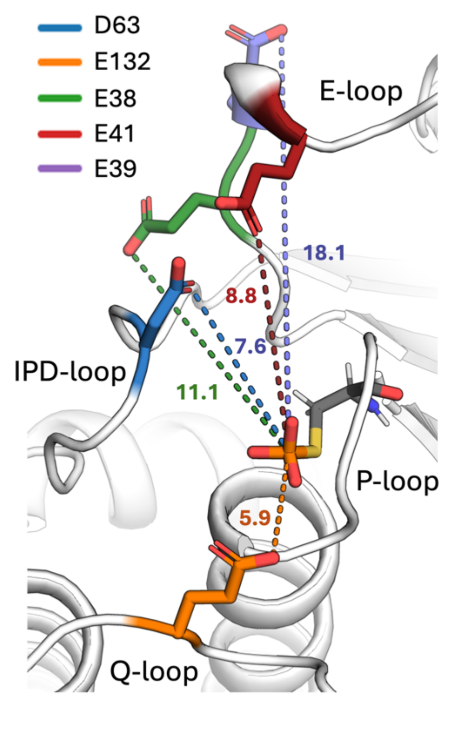

Figure S3. Prospective catalytic backups in the ShufPTP active site. Prospective catalytic backup sidechains in the ShufPTP active site were identified based on the distance between the closest oxygen atom of the side chain and the phosphorus atom of the phosphoenzyme intermediate (which was modelled manually into the structure) highlighted, in the crystal structure of wild-type ShufPTP (PDB ID: 9E9N, this work) Note that although the E-loop residues E38 and E39 clearly point away from the active site in the crystal structure of ShufPTP, we nevertheless included these side chains in our analysis, in case rearrangement of the E-loop could bring these residues closer to the active site during our molecular dynamics simulations.

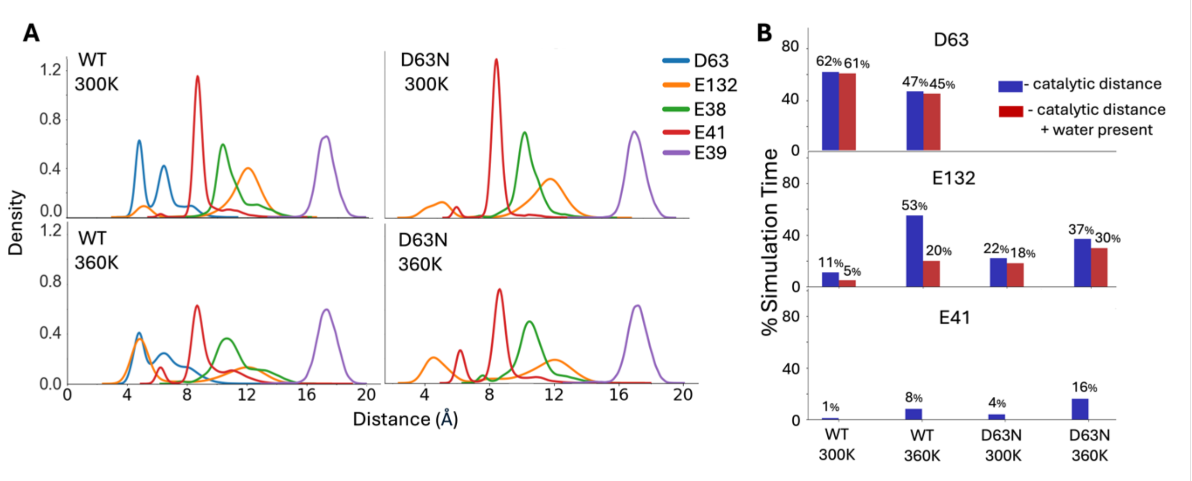

Figure S4. Identifying prospective catalytic backups in the ShufPTP active site. Shown here are the distances between the phosphate group and prospective catalytic carboxylate side chains, extracted from our MD simulations of wild-type and D63N ShufPTP, at the respective phosphoenzyme intermediate states. Simulations were performed at both 300 and 360K. (**A**) Kernel density estimates (KDE) of distances between the phosphate group and prospective catalytic side chains, defined as the distance between the P atom of the phosphate group and the closest oxygen atom of the carboxylate side chain of each key residue, were compared to identify a backup catalytic residue. (**B**) The % simulation time the side chains of each of the three most likely candidates (D63, E41 and E132) spent within 6Å of the phosphate group phosphorus atom (shown in blue). Further, to be catalytically viable, it is necessary for a water molecule to bridge the respective side chain and the phosphate group, in order to act as a nucleophile (**Figure 1**). For each prospective catalytic side chain, we calculated the % of simulation time a water molecule is positioned within both 3.5Å of the carboxylic acid of the respective side chain, and of the phosphorus atom of the phosphate group (red, measured based on distances to the nucleophilic oxygen atom). This panel shows that even though there is a small peak at smaller distances for the E41 side chain in panel A, this side chain is essentially never sampling a catalytic conformation on these simulation timescales, and thus the E41 side chain was ruled out from further consideration.

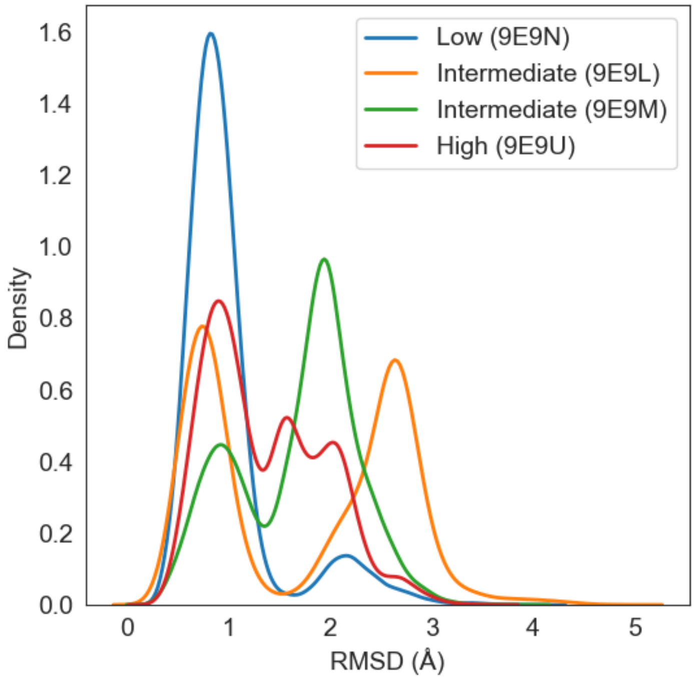

Figure S5. Geometric stability of the R99 side chain. The root means square deviation (RMSD, Å) of the R99 side chain in the simulations of unliganded ShufPTP, initialized from all four crystal structures of ShufPTP presented in this work (**Table S1**). The low P-loop crystal structure was chosen as a common reference for all RMSD measurements. While all simulations show some stabilization of the R99, side chain, which is a key residue required for phosphate binding, only simulations of the "low" crystal structure shows the conformation captured in crystal structures as highly preferred.

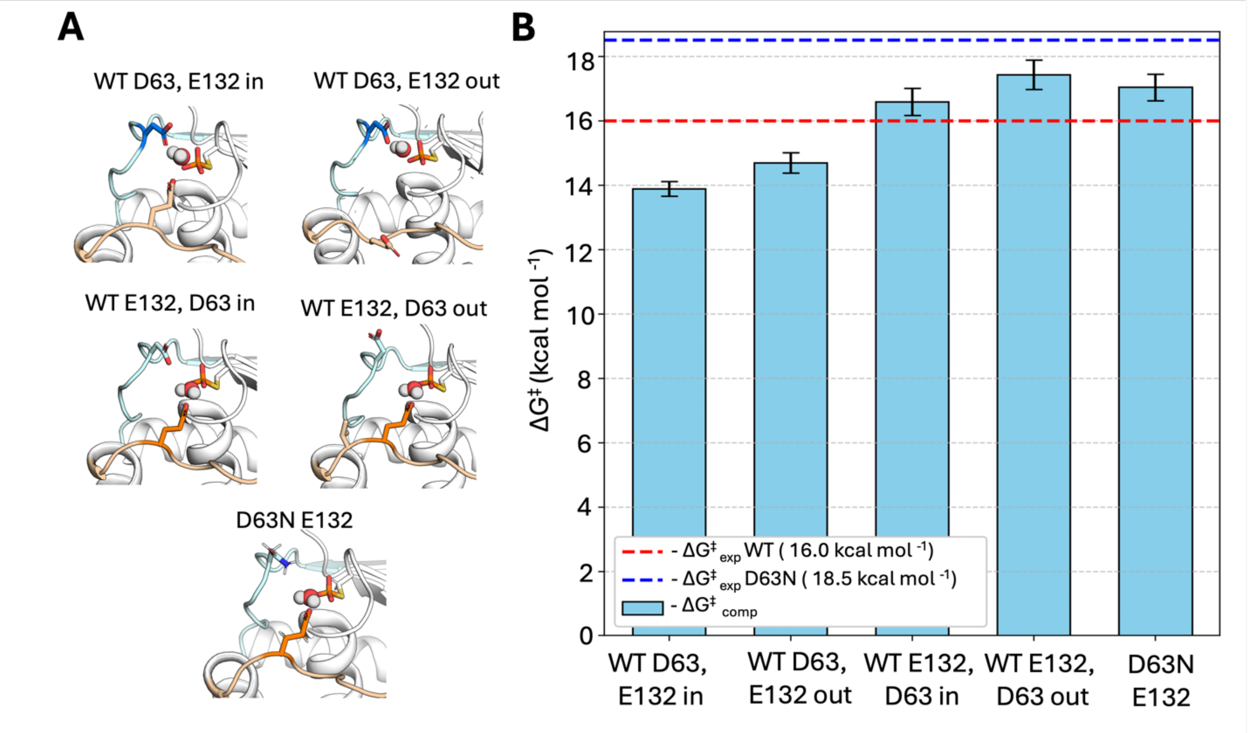

Figure S6. Active site plasticity and backup catalytic mechanisms in ShufPTP. (A) Illustration of the reaction environment in five different catalytic scenarios that were simulated using the empirical valence bond (EVB) approach,[17] to evaluate energy barrier of the reaction facilitated by proton transfer to the D63 or E132 side chains in ShufPTP, and the E143 side chain in the D63N variant**.** In these scenarios, either or both of the D63 and E132 side chains can be pointing into ("in") or out ("out") from the ShufPTP active site. **(B)** The calculated $\Delta G^{\ddagger}$ values (kcal mol^{-1}) for each scenario were compared to the experimental values obtained from the kinetic data (k_{cat}, **Table 1**) for both variants. The red dashed line indicates the experimental activation free energy for the reaction catalyzed by ShufPTP, and the blue dashed line indicates the experimental activation free energy for the reaction catalyzed by the D63N variant. The error bars represent the standard error of the mean on the calculated activation free energies over 20 individual EVB trajectories for each system. The raw data for this figure are shown in **Table S2**.

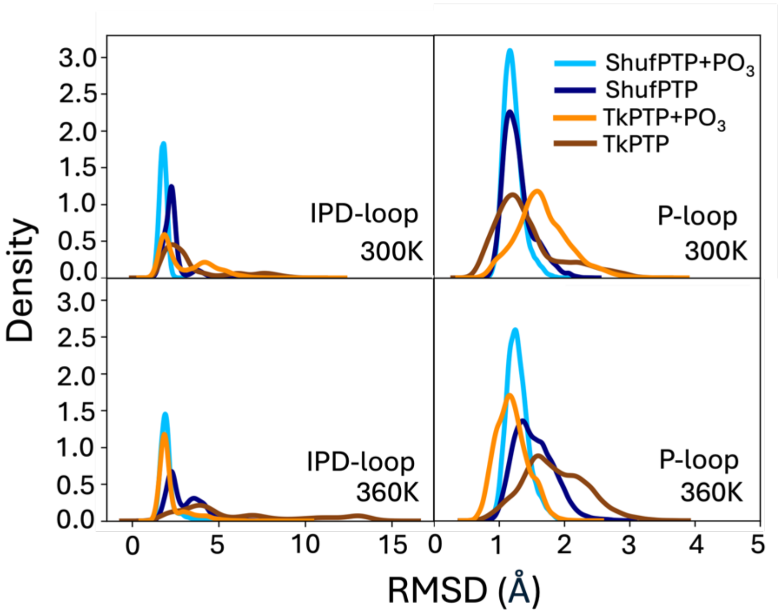

Figure S7. **Displacement the IPD- and P-loops of ShufPTP and TkPTP, during MD simulations of active(low) state**. Kernel density estimation (KDE) analysis of the root mean square deviations (RMSD, Å) for the backbone atoms of the P-and IPD-loops in simulations of TkPTP in its active P-loop state and ShufPTP in the low P-loop state. Shown here are data for both unliganded enzymes and the phosphoenzyme intermediate states (+PO_3), at 300 and 360K. At the phosphoenzyme intermediate state, the conformational ensembles of the IPD- and P-loops shift closer to the respective P-loop active state crystal structure (PDB IDs: 5Z5A[2] and 9E9N), especially in the case of ShufPTP. When the simulation temperature is increased to 360K a greater flexibility of both loops is observed in the unphosphorylated systems. However, in the case of the phosphorylated system the stability of TkPTP catalytic conformation is increased.

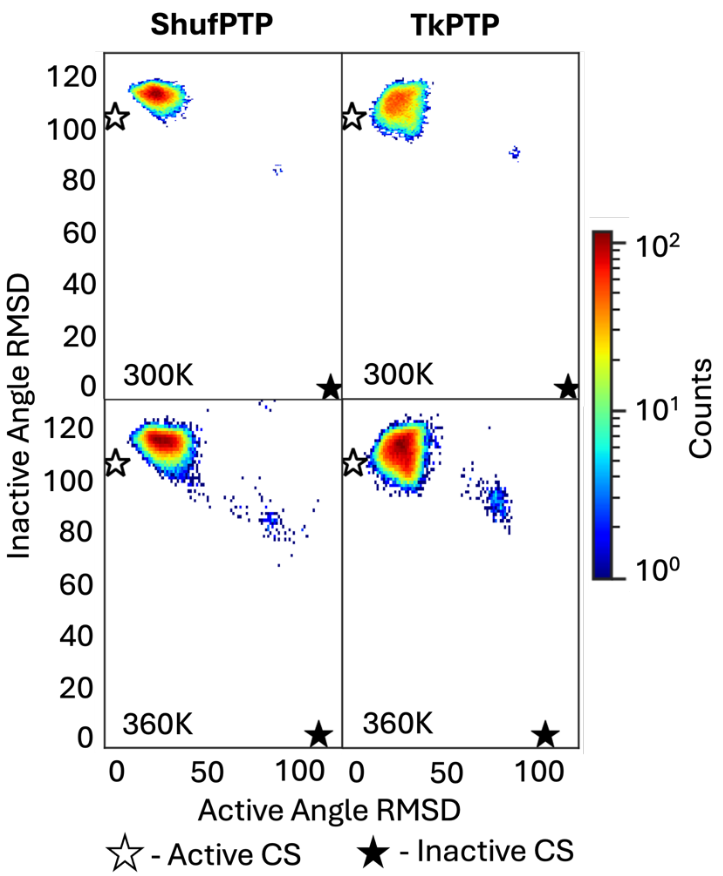

Figure S8. **Comparison of P-loop conformational transitions between ShufPTP and TkPTP, in simulations initialized from the active (low) conformation of the P-loop**. Shown here are 2D histograms of the P-loop conformation sampled in our MD simulations of each enzyme. The deviation between active and inactive state X-ray crystal structures (CS) of ShufPTP and TkPTP corresponds to φ and ψ angles of 116.1 degrees and 104.7 degrees respectively. Simulations were initiated from the active (low) state of each enzyme, with the IPD-loop in its loop-closed state. The corresponding crystallographic positions of the P-loop are denoted by stars, as annotated on the figure.

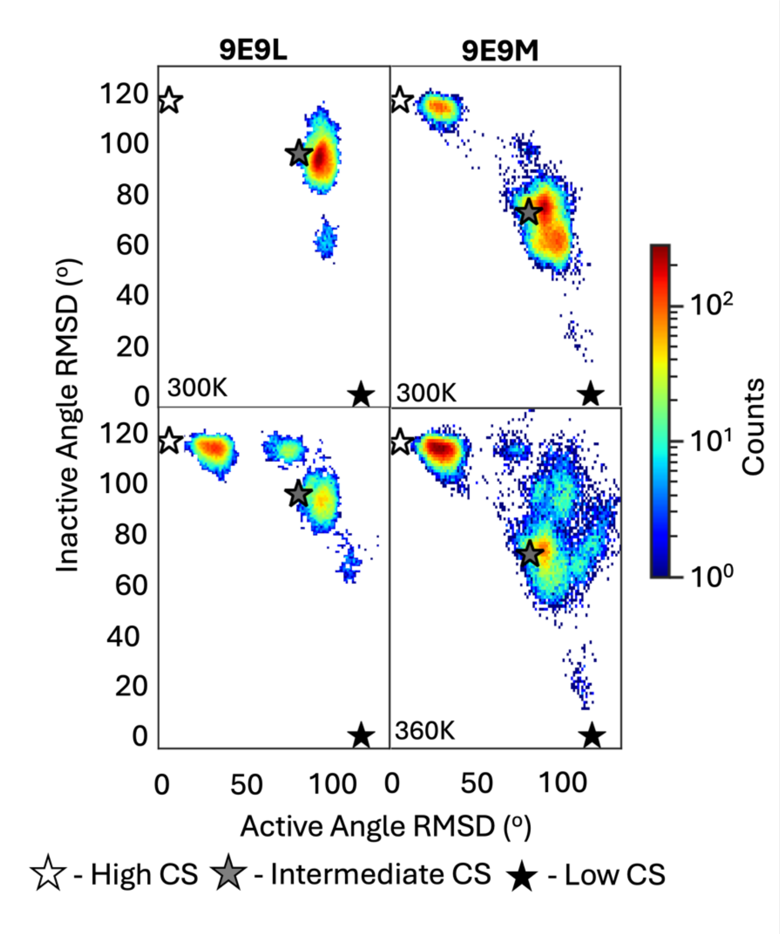

Figure S9. **Comparison of P-loop conformational transitions between ShufPTP, in simulations initialized from the intermediate conformations of the P-loop**. Shown here are 2D histograms of the P-loop conformation sampled in our MD simulations of each enzyme. The deviation between low and high state X-ray crystal structures (CS) of ShufPTP correspond to φ and ψ angles of 116.1 degrees. The angle RMSD of the two ShufPTP intermediate to active (low) and inactive (high) CS are: 9E9L = (78.6, 95.2) and 9E9M = (78.6, 71.6) degrees. Simulations were initiated from both experimentally obtained crystalized intermediate states of P-loop conformation of ShufPTP (PDB ID: 9E9L and 9E9M), with the IPD-loop in its loop-closed state. The corresponding crystallographic positions of the P-loop are denoted by stars, as annotated on the figure.

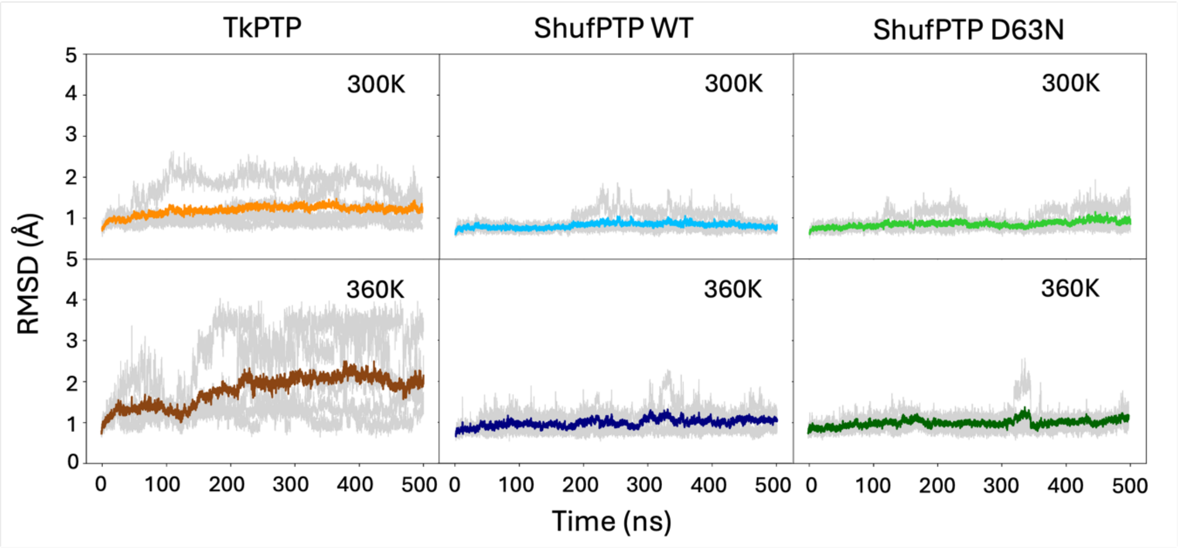

Figure S1. Comparison of backbone root mean square deviations (RMSD, Å) of molecular dynamics simulations of wild-type TkPTP, ShufPTP and the ShufPTP D63N variant, initialized from the unliganded active (low) P-loop crystal structure of ShufPTP at 300K and 360K (PDB IDs: 5Z5A,[2] 9E9N).

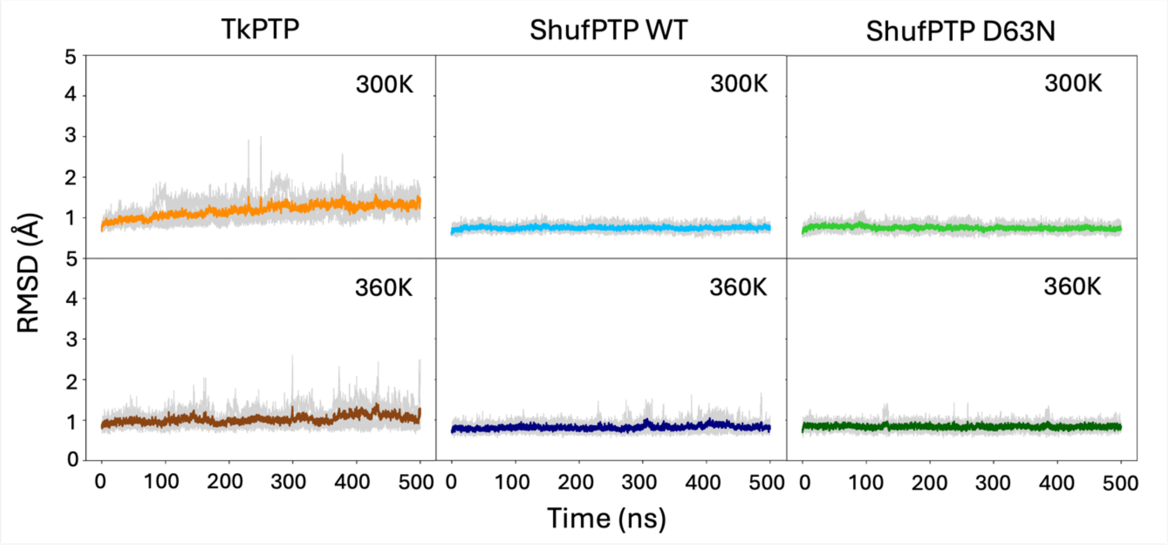

Figure S11. Comparison of backbone root mean square deviations (RMSD, Å) of molecular dynamics simulations of wild-type TkPTP, ShufPTP and the ShufPTP D63N variant, initialized from the active (low) P-loop crystal structure of ShufPTP with phosphorylated C93, at 300K and 360K (PDB IDs: 5Z5A,[2] 9E9N).

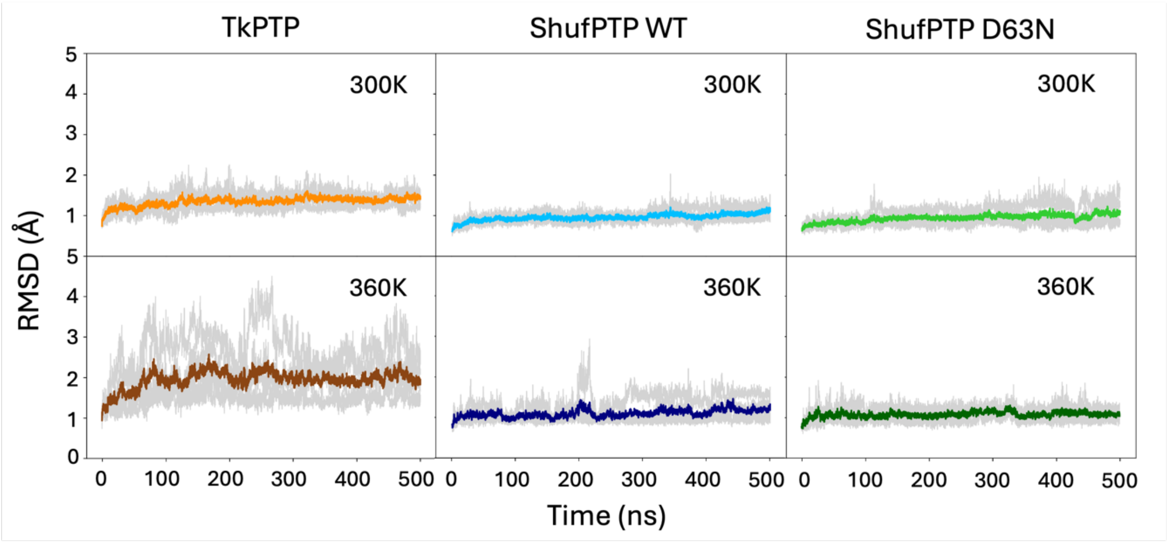

Figure S12. Comparison of backbone root mean square deviations (RMSD, Å) of molecular dynamics simulations of wild-type TkPTP, ShufPTP and the ShufPTP D63N variant , initialized from the unliganded inactive (high) P-loop crystal structure of ShufPTP at 300K and 360K (PDB IDs: 5Z59,[2] 9E9U).

S3. Supplementary Tables

Table S1. X-ray data collection and refinement statistics.

PDB ID	9E9N	9E9M	9E9L	9E9U
P-loop Conformation	Low	Intermediate	Intermediate	High
C93 Oxidation State	Sulfenic Acid	Sulfenic Acid<br>Sulfenamide	Sulfenic Acid	Sulfenic Acid<br>Sulfonic Acid
Ligand	PEG	Sulfate	Vanadate	Vanadate
**Data collection**				
Source	Home Source	Home Source	SSRL 9-2	SSRL 9-2
Wavelength (Å)	1.5418	1.5418	0.97946	0.97946
Resolution range (Å)	50.0-1.94 (2.01-1.94)*	50.0-1.55 (1.61-1.55)	50.0-2.10 (2.18-2.10)	50.0-1.80 (1.87-1.80)
Space group	$P\ 6_1$	$P\ 6_1$	$P\ 6_1$	$P\ 6_1$
Unit cell *a*, *b*, *c* (Å)	85.64, 85.64, 32.2	86.00, 86.00, 32.2	85.72, 85.72, 32.37	86.05, 86.05, 32.23
α, β, γ (°)	90, 90, 120	90, 90, 120	90, 90, 120	90, 90, 120
Total reflections	182280	385923	112232	213691
Unique reflections	10023 (860)	19687 (1907)	7896 (669)	12709 (1148)
Redundancy	18.1 (4.9)	19.6 (16.5)	14.2 (11.1)	16.8 (6.6)
Completeness (%)	98.3 (84.7)	98.2 (95.2)	96.9 (83.5)	98.9 (91.3)
Mean $I/\sigma(I)$	26.5 (1.4)	51.3 (5.9)	15.53 (5.5)	17.0 (2.1)
Wilson B-factor	34.6	20.8	34.1	25.5
CC1/2	(0.575)	(0.942)	(0.942)	(0.743)
**Refinement**				
No. of reflections	10021 (860)	19687 (1907)	7893 (670)	12709 (1148)
R_{work}/R_{free}	0.178/0.231 (0.330/0.471)	0.185/0.211 (0.244/0.334)	0.184/0.224 (0.222/0.234)	0.178/0.214 (0.271/0.328)
No. of atoms				
Protein	1199	1219	1190	1221
Ligand/ion	13	10	29	5
Water	43	129	18	52
B-factors				
Protein	76.7	85.0	37.0	76.7
Ligand/ion	90.6	–	55.2	90.6
Water	47.0	61.1	42.4	47.0
R.m.s. deviations				
Bond lengths (Å)	0.011	0.011	0.011	0.011
Bond angles (°)	1.196	1.196	1.196	1.196
Ramachandran plot				
Favored (%)	89.4	93.4	89.4	89.4
Allowed (%)	9.5	6.1	9.5	9.5
Outliers (%)	1.0	0.4	1.0	1.0
Rotamer outliers (%)	0.82	0.00	5.65	3.88
Clashscore	4.97	7.71	7.45	2.84

*Values in parentheses are for highest-resolution shell.

Table S2. A comparison of experimental and calculated activation free energies of the hydrolysis of the phosphoenzyme intermediate in the reaction catalyzed by ShufPTP and its D63N variant.[a]

System	$\Delta G^{\ddagger}_{calc}$	$\Delta G^{\ddagger}_{exp}$
ShufPTP		
**D63-as-base, E132 in**	13.9 ± 0.2	16.0
**D63-as-base, E132 out**	14.7 ± 0.3	
**E132-as-base, D63 in**	16.6 ± 0.4	
**E132-as-base, D63 out**	17.4 ± 0.4	
**D63N ShufPTP**		
**E132-as-base**	17.0 ± 0.4	18.5

[a] All calculated energies ($\Delta G^{\ddagger}_{calc}$) are presented as average values and standard errors of the mean across 20 independent EVB trajectories, obtained as described in the Materials and Methods. "In" and "out" refer to whether a given side chain is pointing into or out of the active site (**Figure S6**). Note that the catalytic residue is always pointing into the active site. Experimental activation energies ($\Delta G^{\ddagger}_{calc}$) are calculated based on turnover numbers (k_{cat}, s^{-1}) presented in **Table 1**, using transition state theory. In the case of ShufPTP, the lowest energy pathways for each of the D63-as-base and E132-as-base mechanisms are highlighted in blue.

Table S3. List of ionized residues and histidine protonation states in EVB simulations of ShufPTP.[a]

Type	Residue Number
**Asp**	63
**Glu**	22, 38, 39, 41, 132, 135, 138
**Arg**	6, 16, 99, 122, 124, 127
**His-ε**	58, 92

[α] All other ionizable residues not specified in the table were simulated in their neutral state during the simulations, as they fell outside the explicit solvent sphere. His-ε corresponds to a histidine singly protonated on its $N_{\epsilon 2}$ nitrogen atom.

S4. Supplementary References

(1) van der Spoel, D.; Lindahl, E.; Hess, B.; Groenhof, G.; Mark, A. E.; Berendsen, H. J. C. GROMACS: Fast, Flexible and Free. *J. Comp. Chem.* **2005**, *26*, 1701-1718.

(2) Yun, H.-Y.; Lee, J.; Kim, H.; Ryu, H.; Shin, H.-C.; Oh, B.-H.; Ku, B.; Kim, S. J. Structural Study Reveals the Temperature Dependent Conformational Flexibility of Tk-PTP, a Protein Tyrosine Phosphatase from *Thermococcus Kodakaraensis* KOD1. *PLoS One* **2018**, *13*, e0197635.

(3) Huang, J.; Rauscher, S.; Nawrocki, G.; Ran, T.; Feig, M.; de Groot, B. L.; Grubmüller, H.; MacKerell Jr., A. D. CHARMM36m: An Improved Force Field for Folded and Intrinsically Disordered Proteins. *Nat. Methods.* **2016**, *14*, 71-73.

(4) Jorgensen, W. L.; Chandrasekhar, J.; Madura, J. D.; Impey, R. W.; Klein, M. L. Comparison of Simple Potential Functions for Simulating Liquid Water. *J. Chem. Phys.* **1983**, *79*, 926-935.

(5) Jo, S.; Kim, T.; Iyer, V. G.; Im, W. CHARMM-GUI: A Web-based Graphical User Interface for CHARMM. *J. Comput. Chem.* **2008**, *29*, 1859-1865.

(6) Bussi, G.; Donadio, D.; Parrinello, M. Canonical Sampling Through Velocity Rescaling. *J. Chem. Phys.* **2007**, *126*, 014101.

(7) Bernetti, M.; Bussi, G. Pressure Control Using Stochastic Cell Rescaling. *J. Chem. Phys.* **2020**, *153*, 144107.

(8) Hess, B.; Bekker, H.; Berendsen, H. J. C.; Fraaije, J. G. E. M. LINCS: A Linear Constraint Solver For Molecular Simulations. *J. Comp. Chem.* **1997**, *18*, 1463-1472.

(9) Grubmüller, H.; Heller, H.; Windemuth, A.; Schulten, K. Generalized Verlet Algorithm for Efficient Molecular Dynamics Simulations with Long-range Interactions. *Mol. Sim.* **1990**, *6*, 121-142.

(10) Darden, T.; York, D.; Pedersen, L. Particle Mesh Ewald: An *N*·log(*N*) Method for Ewald Sums in Large Systems. *J. Chem. Phys.* **1993**, *98*, 10089-10092.

(11) Gowers, R.; Linke, M.; Barnoud, J.; Reddy, T.; Melo, M.; Seyler, S.; Domanski, J.; Dotson, D.; Buchoux, S.; Kenney, I.; et al. MDAnalysis: A Python Package for the Rapid Analysis of Molecular Dynamics Simulations. *Proc. 15th Python Sci. Conf.* **2016**.

(12) Michaud-Agrawal, N.; Denning, E. J.; Woolf, T. B.; Beckstein, O. MDAnalysis: A Toolkit for the Analysis of Molecular Dynamics Simulations. *J. Comp. Chem.* **2011**, *32*, 2319-2327.

(13) Theobald, D. L. Rapid Calculation of RMSD Using a Quaternion-Based Characteristic Polynomial. *Acta Cryst. A* **2005**, *61*, 478-480.

(14) Liu, P.; Agrafiotis, D. K.; Theobald, D. L. Fast Determination of the Optimal Rotational Matrix for Macromolecular Superpositions. *J. Comp. Chem.* **2009**, *31*, 1561-1563.

(15) The PyMOL Molecular Graphics System. Version 3.0. Schrödinger, LLC.

(16) Yehorova, D.; Crean, R. M.; Kasson, P. M.; Kamerlin, S. C. L. Key Interaction Networks: Identifying Evolutionarily Conserved Non-Covalent Interaction Networks Across Protein Families. *Prot. Sci.* **2024**, *33*, e4911.

(17) Warshel, A.; Weiss, R. M. An Empirical Valence Bond Approach for Comparing Reactions in Solutions and in Enzymes. *J. Am. Chem. Soc.* **1980**, *102*, 6218-6226.

(18) Warshel, A.; King, G. Polarization Constraints in Molecular Dynamics Simulation of Aqueous Solutions: The Surface Constraint All Atom Solvent (SCAAS) Model. *Chem. Phys. Lett.* **1985**, *121*, 124-129.

(19) Crean, R. M.; Biler, M.; van der Kamp, M. W.; Hengge, A. C.; Kamerlin, S. C. L. Loop Dynamics and Enzyme Catalysis in Protein Tyrosine Phosphatases. *J. Am. Chem. Soc.* **2021**, *143*, 3830-3845.

(20) Shen, R.; Crean, R. M.; Olsen, K. J.; Corbella, M.; Calixto, A. R.; Richan, T.; Brandão, T. A. S.; Berry, R. D.; Tolman, A.; Loria, J. P.; et al. Insights into the Importance of WPD-Loop Sequence in Protein Tyrosine Phosphatases. *Chem. Sci.* **2022**, *13*, 13524-13540.

(21) Crean, R.; Corbella, M.; Calixto, A. R.; Hengge, A.; Kamerlin, S. C. L. Sequence – Dynamics – Function Relationships in Protein Tyrosine Phosphatases. *QRB Discov.* **2024**, *5*, e4.

(22) Crean, R. M.; Biler, M.; Corbella, M.; Calixto, A. R.; van der Kamp, M.; Hengge, A. C.; Kamerlin, S. C. L. Correction to "Loop Dynamics and Enzyme Catalysis in Protein Tyrosine Phosphatases". *J. Am. Chem. Soc.* **2022**, *144*, 10091-10093.

(23) Bauer, P.; Barrozo, A.; Purg, M.; Amrein, B. A.; Esguerra, M.; Wilson, P. B.; Major, D. T.; Åqvist, J.; Kamerlin, S. C. L. Q6: A Comprehensive Toolkit for Empirical Valence Bond and Related Free Energy Calculations. *SoftwareX* **2018**, *7*, 388-395.

(24) Jorgensen, W. L.; Maxwell, D. S.; Tirado-Rives, J. Development and Testing of the OPLS All-Atom Force Field on Conformational Energetics and Properties of Organic Liquids. *J. Am. Chem. Soc.* **1996**, *118*, 11225-11236.

(25) Lee, F. S.; Warshel, A. A Local Reaction Field Method for Fast Evaluation of Long-range Electrostatic Interactions in Molecular Simulations. *J. Chem. Phys.* **1992**, *97*, 3100-3107.

(26) Berendsen, H. J. C.; Postma, J. P. M.; van Gunsteren, W. F.; DiNola, A.; Haak, J. R. Molecular Dynamics with Coupling to an External Bath. *J. Chem. Phys.* **1984**, *81*, 3684-3690.

(27) Clark, K.; Karsch-Mizrachi, I.; Lipman, D. J.; Ostell, J.; Sayers, E. W. GenBank. *Nucleic Acids Res.* **2016**, *44*, D67-D72.
